# Discovery of Stealthin Derivatives and Implication of the Amidotransferase FlsN3 in the Biosynthesis of Nitrogen-Containing Fluostatins

**DOI:** 10.3390/md17030150

**Published:** 2019-03-04

**Authors:** Chunshuai Huang, Chunfang Yang, Zhuangjie Fang, Liping Zhang, Wenjun Zhang, Yiguang Zhu, Changsheng Zhang

**Affiliations:** 1 CAS Key Laboratory of Tropical Marine Bio-resources and Ecology, Guangdong Key Laboratory of Marine Materia Medica, RNAM Center for Marine Microbiology, Institutions of South China Sea Ecology and Environmental Engineering, South China Sea Institute of Oceanology, Chinese Academy of Sciences, 164 West Xingang Road, Guangzhou 510301, China; 15969592400@163.com (C.H.); chunfangy@126.com (C.Y.); fangzhuangjie16@mails.ucas.ac.cn (Z.F.); zhanglp@scsio.ac.cn (L.Z.); wzhang@scsio.ac.cn (W.Z.); zhuyiguang2003@163.com (Y.Z.); 2 University of Chinese Academy of Sciences, 19 Yuquan Road, Beijing 100049, China

**Keywords:** angucyclines, N–N bond, raceme, gene inactivation, biosynthesis, marine, *Micromonospora*

## Abstract

Diazobenzofluorene-containing atypical angucyclines exhibit promising biological activities. Here we report the inactivation of an amidotransferase-encoding gene *flsN3* in *Micromonospora rosaria* SCSIO N160, a producer of fluostatins. Bioinformatics analysis indicated that FlsN3 was involved in the diazo formation. Chemical investigation of the *flsN3*-inactivation mutant resulted in the isolation of a variety of angucycline aromatic polyketides, including four racemic aminobenzo[*b*]fluorenes stealthins D–G (**9**–**12**) harboring a stealthin C-like core skeleton with an acetone or butanone-like side chain. Their structures were elucidated on the basis of nuclear magnetic resonance (NMR) spectroscopic data and X-ray diffraction analysis. A plausible mechanism for the formation of stealthins D–G (**9**–**12**) was proposed. These results suggested a functional role of FlsN3 in the formation/modification of N–N bond-containing fluostatins.

## 1. Introduction

**Figure 1:**
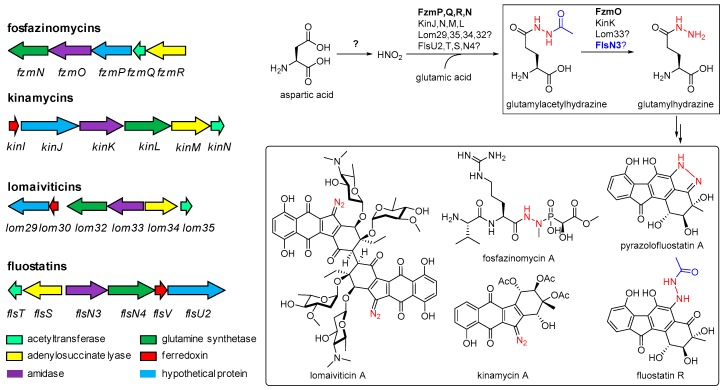
Conserved enzymes and their proposed roles in N–N bond formation and modification in the biosynthetic pathways of fosfazinomycins, kinamycins, lomaiviticins, and fluostatins.

A growing number of benzofluorene-containing atypical angucycline type of aromatic polyketides have been discovered to exhibit significant antibacterial and antitumor activities [1,2], including kinamycins [3], lomaiviticins [4,5,6], nenestatins [7], and fluostatins [8,9,10,11,12,13]. The diazo groups in lomaiviticin A have been reported to play an indispensable role in the potent cytotoxicity against a panel of human cancer cell lines by inducing double-strand breaks in DNA via the formation of vinyl radical intermediate [4,6]. The diazo group was also present in structually diverse natural products with promising biological activities, such as cremeomycin [14], and kinamycins [15,16]. Biosynthesis of the diazo group in cremeomycin was recently unveiled to invovle the direct coupling of a nitrous acid with the primary amino acid in a biosynthetic precursor [14,17]. One of the nitrogen atoms in the diazo group of kinamycins was reported to also be derived from the nitrous acid; however, the N–N bond was revealed to be first formed from aspartic acid as a discrete and separate synthon, and then was transferred to the scaffold using glutamic acid as carrier, the same as that in fosfazinomycins (Figure 1) [18,19].

The enzymes for the N–N bond formation and modification in fosfazinomycins (FzmN, FzmO, FzmP, FzmQ, and FzmR) and kinamycins (KinI, KinJ, KinK, KinL, KinM and KinN) are also conserved in lomaiviticin pathways (Lom29, Lom30, Lom32, Lom 33, Lom34, and Lom35), indicating a similar fashion to assembly the N–N bond in lomaiviticins (Figure 1) [20]. Interestingly, homologues of the conserved diazo forming enzymes are also present in the biosynthetic pathway of fluostatins (FlsT, FlsS, FlsN3, FlsN4, FlsV, and FlsU2, Figure 1) in the marine derived *Micromonospora rosaria* SCSIO N160 [9], although the dominant fluostatin products do not contain a diazo group [9]. However, a group of N–N bond-containing compounds have been identifed in a minor amount from *M. rosaria* SCSIO N160 and the heterologous host *Streptomyces albus* J1074 expressing the fluostatin biosynthetic gene cluster (*fls*), including pyrazolofluostatin A [10], and fluostatin R (Figure 1) [12]. The identification of these compounds indicated that the set of N–N bond forming enzymes should be functional in the fluostatin biosynthetic pathway. To find out whether these enzymes were truly involved in the N–N bond formation and modification, the *flsN3* gene, putatively encoding an amidotransferase homologous to KinK, FzmO and Lom33 (Figure 1), was disrupted by insertional mutagenesis. Here we reported the isolation and structural elucidation of four new racemic aminobenzo[*b*]fluorenes stealthins D–G from the *flsN3* inactivation mutant, and the implication of FlsN3 in fluostatin biosynthesis.

## 2. Results and Discussion

### 2.1. Gene Inactivation and Compound Isolation

**Figure 2:**
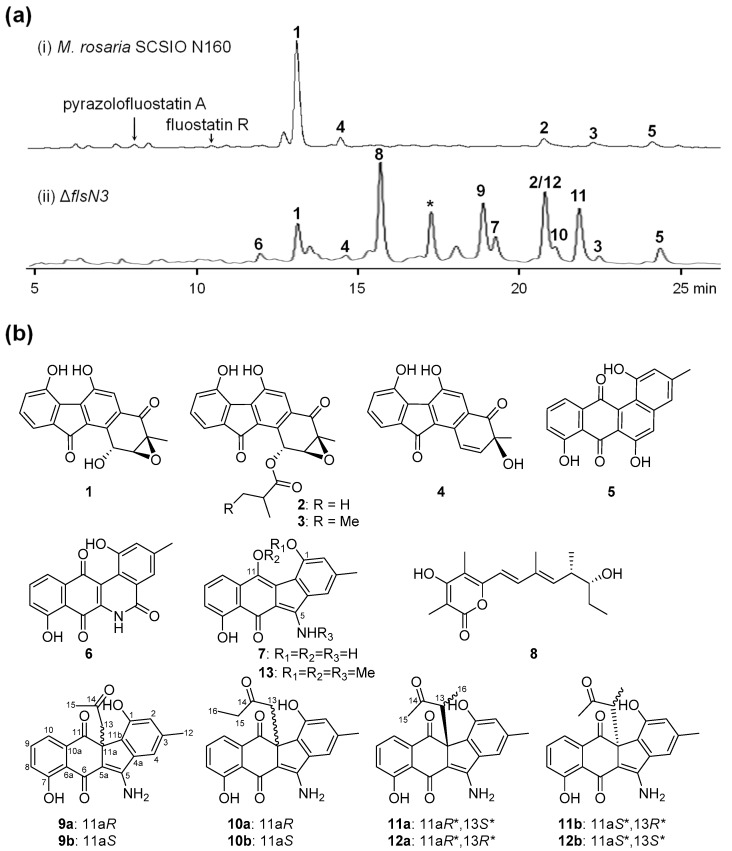
Secondary metabolites from the Δ*flsN3* mutant. (**a**) HPLC analysis of the metabolite profiles of the wild type strain and the Δ*flsN3* mutant with the detection wavelength at 354 nm; the N–N bond-containing products (pyrazolofluostatin A and fluostatin R, Supplementary Figure S2) were marked; the uncharacterized compound denoted with an asterisk was related to salinipyrone A (**8**). (**b**) Chemical structures of compounds isolated from the Δ*flsN3* mutant.

Bioinformatics analysis showed that *flsN3* encoded a glutamyl-tRNA(Gln) amidotransferase subunit A that exhibited high amino acid identity to KinK (66%), Lom33 (66%), and FzmO (52%). FzmO and KinK were recently shown to be related to the N–N bond formation in fosfazinomycins and kinamycins, respectively. To further probe the function of *flsN3*, a conventional PCR-targeting-based insertional mutagenesis was employed to construct the Δ*flsN3* mutant (Supplementary Figure S1 and Table S1). HPLC (high performance liquid chromatography) analysis revealed that the Δ*flsN3* mutant showed a metabolite profile different from that of the wild type strain *M. rosaria* SCSIO N160 (Figure 2a).

Subsequently, a total of 11 FST-related compounds were isolated from 14 L cultures of the Δ*flsN3* mutant, including seven known compounds fluostatins C, D, J, and K (**1**–**4**) [8], dehydrorabelomycin (**5**) [9], benzo[*b*]phenanthridone (**6**) [21], stealthin C (**7**) [22], and four pair of new racemic aminobenzo[*b*]fluorenes stealthins D–G (**9**–**12**, Figure 2b, Supplementary Figure S2). In addition, salinipyrone A (**8**) was isolated from the Δ*flsN3* mutant (Figure 2b), and the production of an analogue related to salinipyrone A (**8**) was also observed (the compound denoted with an asterisk in trace ii, Figure 2a, and Supplementary Figure S3). In accordance with the finding that salinipyrones were biosynthetic byproducts of the rosamicin polyketide synthase (PKS) in the marine actinomycete *Salinispora pacifica* [23,24]. *M. rosaria* SCSIO N160 was previously shown as a rosamicin producer [25]. Therefore, we speculated that the overproduction of salinipyrone A (**8**) might be due to the fact that the inactivation of *flsN3* triggered an unknown mechanism to cause module skipping in rosamicin PKS.

### 2.2. Structural Elucidation

Stealthin C (**7**) was known to be NMR silent due to the presence of mixed oxidation states, including a free radical under ambient conditions [22,26,27], we therefore treated **7** with methyl iodide to yield a trimethyl derivative that was subsequently characterized as trimethylstealthin C (**13**, Supplementary Figure S4), thus, compound **7** was confirmed to be stealthin C. Compounds **9**–**12** were found to be four new stealthin C-related racemic mixtures of aminobenzo[*b*]fluorenes (Figure 2b).

**Figure 3:**
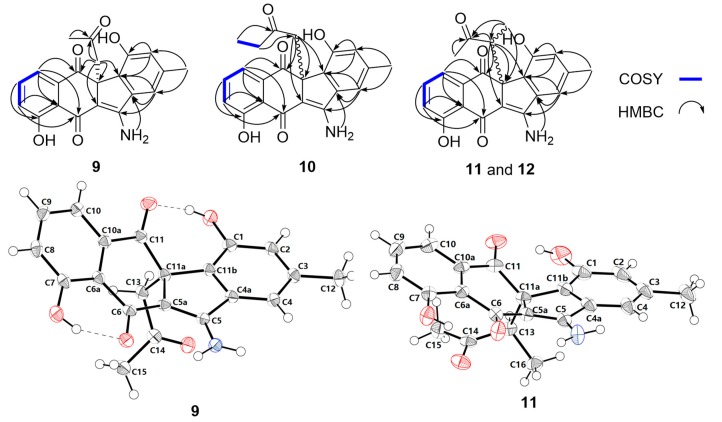
Key COSY and HMBC correlations of **9**–**12**, and single crystal X-ray diffractions of **9** and **11**.

Stealthin D (**9**) was obtained as a yellow crystal. The molecular formula of **9** was established as C_21_H_17_NO_5_ by high-resolution electrospray ionization mass spectrometry (HRESIMS) (*m*/*z* 364.1183 [M + H]^+^, calcd 364.1179, Supplementary Figure S5). Analysis of the ^1^H NMR spectrum of **9** (Table 1, Supplementary Figure S6) revealed the presence of two singlet methyls (*δ*_H_ 1.71, 2.33), a methylene (two coupled proton signals at *δ*_H_ 2.87, d, *J* = 13.7; *δ*_H_ 3.40, d, *J* = 13.7), and five olefinic protons (including a group of characteristic aromatic ABC spin system *δ*_H_ 7.17/7.55/7.23). In addition, the four downfield singlet proton signals (*δ*_H_ 8.12, 8.55, 9.69, and 13.32) might be attributed to amine or hydroxyl group protons. The ^13^C NMR spectrum of **9** (Table 2, Supplementary Figure S7) exhibited 21 carbon signals, which were classified by DEPT-135 experiment as two methyls, one methylene, five olefinic methines, and 13 quaternary carbons including nine olefinic carbons and three ketos (*δ*_C_ 182.7, 196.6, and 204.1). Detailed analyses of the 2D NMR data of **9** (Figure 3, Supplementary Figures S8–S10) revealed that the skeleton of **9** was very similar to that of stealthin C (**7**) [22,26]. The C-11 hydroxyl group in stealthin C (**7**) was changed to a keto group in **9**, which was supported by the HMBC correlations from H-10 and H_2_-13 to C-11 (Figure 3 and Supplementary Figure S10). In addition, a 2-oxo-propyl group was found in **9**, which was located at C-11a by HMBC correlations from H_3_-15 and H_2_-13 to C-14 and from H_2_-13 to C-5a/C-11/C-11a/C-11b. Thus, the planar structure of **9** was established. It was noteworthy that **9** showed ignorable optical rotation (${\left[\alpha \right]}_{D}^{25}$ − 2.3; *c* 0.20, MeOH). A chiral-phase HPLC analysis of **9** indicated that it was a pair of racemic mixture of two enantiomers **9a** (11a*R*) and **9b** (11a*S*) (Supplementary Figure S2). Finally, the single crystal X-ray diffraction analysis completely elucidated the structure of **9** and confirmed the racemic nature of **9** (CCDC no. 1887925; Figure 3, Supplementary Table S2).

The molecular formula of stealthin E (**10**) was determined to be C_22_H_19_NO_5_ by HRESIMS (*m*/*z* 378.1334 [M + H]^+^, calcd 378.1336, Supplementary Figure S11). Detailed analysis of the NMR data of **10** (Table 1 and Table 2, Supplementary Figures S12−S16) revealed that **10** shared high structure similarity with **9**. The only difference was that a singlet methyl Me-15 (*δ*_H_ 1.71/*δ*_C_ 30.9) was present in **9**, while an ethyl group [*δ*_H_ 0.62 (t, *J* = 7.2 Hz, Me-16), 2.02 (dq, *J* = 18.2, 7.2 Hz, H-15a), 2.12 (dq, *J* = 18.2, 7.2 Hz, H-15b), *δ*_C_ 7.2 (Me-16), 36.3 (C-15)] was found in **10**, which was further supported by the COSY correlation between Me-16 and CH_2_-15 (Figure 3, Supplementary Figure S14). Similar to **9**, stealthin E (**10**) had negligible optical rotation (${\left[\alpha \right]}_{D}^{25}$ + 4.1; *c* 0.20, MeOH). A subsequent chiral-phase HPLC analysis confirmed that compound **10** was a racemic mixture consisting of two enantiomers **10a** (11a*R*) and **10b** (11a*S*) (Supplementary Figure S2).

Stealthin F (**11**) was assigned a molecular formula as C_22_H_19_NO_5_ by HRESIMS (*m*/*z* 378.1334 [M + H]^+^, calcd 378.1336, Supplementary Figure S17). A careful comparison of the 1D and 2D NMR spectroscopic data (Table 1 and Table 2, Supplementary Figures S18−S22) of **11** and **9** revealed that they were remarkably similar. A C-13 methylene was observed in **9**. In contrast, a doublet methyl *δ*_H_ 0.73 (d, *J* = 7.0, Me-16) and a quartet methine proton 3.52 (q, *J* = 7.0, H-13) appeared in **11**. The location of the methyl group at C-13 in **11** was supported by key HMBC correlations from H-16 to C-11a/C-13/C-14 and the COSY correlation between Me-16/H-13 (Figure 3, Supplementary Figures S20 and S22). Thus, the planar structure of **11** was determined as shown in Figure 2b. Stealthin F (**11**) showed little optical rotation (${\left[\alpha \right]}_{D}^{25}$ + 1.3; *c* 0.20, MeOH). A chiral-phase HPLC analysis suggested that compound **11** was also a racemic mixture of two enantiomers (Supplementary Figure S2). Finally, the X-ray diffraction analysis of **11** (CCDC no. 1887926; Figure 3, Table S3) confirmed its planar structure and revealed a relative configuration of 11aS*, 13R*. Thus, **11** was tentatively assigned to be an enantiomeric mixture of **11a** (11aS*, 13R*) and **11b** (11aR*, 13S*).

Stealthin G (**12**) showed a molecular formula of C_22_H_19_NO_5_ by HRESIMS (*m*/*z* 378.1343 [M + H]^+^, calcd 378.1336, Supplementary Figure S23). Careful comparison of the highly similar 1D and 2D NMR spectroscopic data (Table 1 and Table 2, Supplementary Figures S24−S28) of **12** and **11** suggested that they should be diastereomers sharing the same planar structure. Compound **12** showed trivial optical rotation (${\left[\alpha \right]}_{D}^{25}$ + 3.5; *c* 0.20, MeOH) and was revealed to be a pair of enantiomeric mixture by a chiral-phase HPLC analysis (Figure 2, Supplementary Figure S2). Considering that the relative configuration of **11** (11aS*, 13R*) was determined by the X-ray diffraction analysis (Figure 3), stealthin G (**12**) was thus assigned as an enantiomeric mixture of **12a** (11aS*, 13S*) and **12b** (11aR*, 13R*).

### 2.3. Biosynthetic Implications

**Figure 4:**
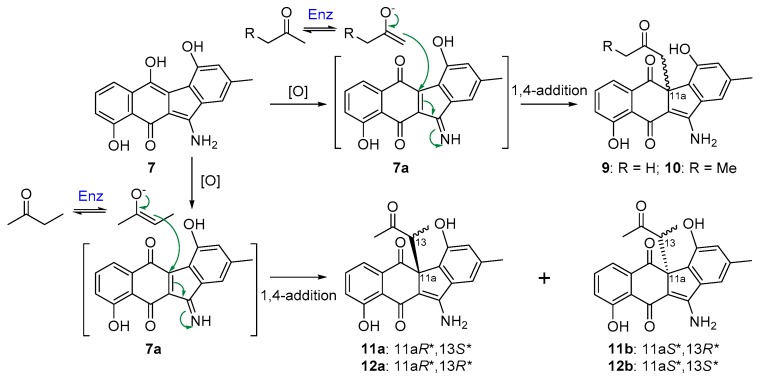
A proposed mechanism for the formation of **9**–**12**.

Inactivation of *flsN3*, encoding a putative N–N bond forming enzyme, led to the Δ*flsN3* mutant that could still produce fluostatins C, D, J, and K (**1**–**4**). However, the production of fluostatin C (**1**), the dominant product in the wild type strain *M. rosaria* SCSIO N160, was dramatically decreased in the Δ*flsN3* mutant (Figure 2). Intriguingly, stealthin C (**7**), and four pair of enantiomeric mixtures stealthins D–G (**9**–**12**) were found to be the major products in the Δ*flsN3* mutant. Stealthin C (**7**) was recently proven as a shunt product in the kinamycin biosynthetic pathway and was shown to be likely synthesized via a nonenzymatic S–N-type Smiles rearrangement [22]. The isolation of racemic mixtures is often an indication of nonenzymatic modifications of natural products. It seems that stealthins D–G (**9**–**12**) are **7** adducts produced during the extraction process using acetone and butanone by reaction with an oxidized **7** species (Figure 4). However, stealthin C (**7**) was found to be quite stable in acetone and butanone under room temperature and it should require additional enzyme to faciliate the formation of **9**–**12** from **7**. It was conceivable that stealthin C (**7**) could undergo spontaneous or enzyme-catalyzed oxidation to produce a dehydrogenated precursor **7a**. Subsequently, the highly reactive alkenol intermediate, probably generated from acetone or butanone by a not-yet-identified dehydrogenase, could act as an electron donor to attack the electrophilic C-11a of the precursor **7a** through 1,4-nucleophilic additions (Figure 4). By this way, the end-products **9**–**12** would be produced as racemic mixtures of enantiomers. In support of this proposed mechanism, enzymes were recently reported to catalyze the conversion of acetone (or butanone) to the alkenol intermediate, such as the acetone carboxylase [28], and the wheat germ lipase (WGL) [29]. In particular, our proposed mechanism was the same as the reported one for the lipase (WGL) [29], which was also quite common and well-known in nature.

Given that none of N–N bond-containing metabolites were observed in the Δ*flsN3* mutant, we speculated that FlsN3 might be involved in the N–N bond modification, by catalyzing the hydrolysis of the acetyl group in glutamylacetylhydrazine to produce glutamylhydrazine, functionally similar to its homologues FzmO and KinK (Figure 1). However, it was still a long way to unveil the unusual biosynthetic machinery to understand how the glutamylhydrazine was incorporated into the N–N bond-containing fluostatins.

## 3. Materials and Methods

### 3.1. General Experimental Procedures

Optical rotation was recorded on a 341 polarimeter (Perkin Elmer, Inc., Norwalk, CT, USA). UV spectrum was obtained with a U-2900 spectrophotometer (Hitachi, Tokyo, Japan). IR spectrum was measured using a Nicolet^∗^6700 FT-IR spectrometer (Thermo Scientific, Waltham, MA USA). 1D and 2D NMR spectroscopic data were obtained on an Avance-500 MHz or an Avance III HD 700 MHz spectrometer (Bruker Biospin GmbH, Rheinstetten, Germany) with tetramethylsilane (TMS, Cambridge Isotopes Laboratories, Inc., Andover, MA, USA) as the internal standard. Low-resolution electrospray ionization mass spectrometric (LRESIMS) and HRESIMS data were collected using an amaZon SL ion trap mass spectrometer and a MaXis 4G UHR-TOFMS spectrometer (Bruker Daltonics Inc., Billerica, MA, USA ), respectively. Thin layer chromatography (TLC,) was performed on precoated silica gel GF_254_ (10–40 μm, Qingdao Marine Chemical Factory, Qingdao, China) glass plates. Silica gel (100–200 mesh, Qingdao Marine Chemical Factory, Qingdao, China), Sephadex LH-20 (40–70 μm; Amersham Pharmacia Biotech AB, Uppsala, Sweden), and YMC*gel ODS-A (12 nm S-50 μm; Kyoto, Japan) were used for column chromatography (CC). Medium pressure liquid chromatography (MPLC) was performed on automatic flash chromatography (CHEETAHTM MP 200, Bonna-Agela Technologies Co., Ltd.,Tianjin, China) with the monitoring wavelength at 254 nm and the collecting wavelength at 280 nm. The analytical HPLC was performed on an Agilent 1260 Infinity series instrument (Agilent Technologies, Inc., Santa Clara, CA, USA) equipped with a diode array detector (DAD) using an Agilent ZORBAX SB-C18 column (150 mm × 4.6 mm, 5 μm) or a Chiral ND 5u (4.6 × 250 mm) chiral column (Phenomenex, Washington, CD, USA) and run the following program: 5% B to 80% B (linear gradient, 0–20 min), 80% B to 100% B (20–21 min), 100% B (isocratic elution, 21–24 min), 100% B to 5% B (24–25 min), 5% B (isocratic elution, 25–30 min); the solvent system comprises solvent A (10% acetonitrile in water supplemented with 0.08% formic acid) and B (90% acetonitrile in water). Semipreparative HPLC was carried out on a Hitachi-L2130 HPLC (Hitachi, Tokyo, Japan) using a Phenomenex Luna C18 column (250 mm × 10 mm, 5 μm).

### 3.2. Construction of the flsN3 Inactivation Mutant

The *flsN3* gene was inactivated by the conventional PCR-targeting method using an apramycin resistance cassette from pIJ773 [30]. The gene cassette comprising *oriT* and *aac(3)IV*, was amplified from plasmid pIJ773 with appropriate primers (Supplementary Table S1) and followed by gel purification. Details for the construction of the cosmid pCSG5028 (Supplementary Table S1) and the insertional inactivation of *flsN3* were described in Supplementary Figure S1. Conjugation between *E. coli* ET12567/pUZ8002/pCSG5028 to *M. rosaria* SCSIO N160 was performed using the following method. Briefly, the cell pellets of *E. coli* ET12567/pUZ8002/pCSG5028 were mixed with mycelia of *M. rosaria* SCSIO N160, and then the mixtures were plated on the ISP4 agar medium. After incubation at 30 °C for 20–24 h, the plates were supplemented with apramycin (100 g mL^−1^) and trimethoprim (TMP, 100 g mL^−1^) for the selection of positive transconjugants.

### 3.3. Fermentation, Extraction, and Isolation

A total of 14 L fermentation cultures of the *∆flsN3* mutant were performed by inoculating 20 mL of the seed culture into to 200 mL of the production medium of modified N4 (0.4% peptone fish, 1% starch soluble, 0.6% corn powder, 0.2% bacterial peptone, 0.5% glycerol, 0.2% CaCO_3_, 3% sea salt, pH 7.0) in a 1 L Erlenmeyer flask, and cultured on a rotary shaker (200 rpm) at 28 ºC for 5 to 7 days. The fermentation cultures were collected and separated to supernatants and mycelia by centrifugation. The mycelia were extracted 3 times with 1.5 L acetone, and the solvent was removed by rotary evaporation. In addition, then the extract of mycelia was mixed with the supernatants and extracted with 14 L butanone for 3 times. The butanone extract was concentrated to dryness, yielding the crude extract (14.5 g). The crude extract was chromatographed over the normal phase silica gel column (100–200 mesh, 20.0 g) and eluted with a gradient solvent system of chloroform/methanol (from 100:0 to 0:100, *v*/*v*) to obtain four fractions (Fr.1 to Fr.4) based on the TLC analysis. Fraction Fr.1 was separated and purified sequentially by C18 reversed-phase MPLC, Sephadex LH-20 column, and semipreparative HPLC to afford **2** (15.0 mg), **3** (12.5 mg), **5** (35.5 mg), **9** (9.0 mg), **10** (5.3 mg), **11** (12.6 mg), and **12** (7.0 mg). Fraction Fr.2 was separated by Sephadex LH-20 column and further purified by semipreparative HPLC to get **7** (10.0 mg) and **8** (2.2 mg). Compounds **1** (21.5 mg), **4** (7.2 mg), and **6** (2.5 mg) were obtained from Fr.3 by reversed-phase MPLC and semipreparative HPLC, successively.

### 3.4. Synthesis of Trimethylstealthin C (13)

The chemical methylation of stealthin C (**7**) was performed according to a previously described method [22,26]. Powdered K_2_CO_3_ (50.0 mg) was slowly added to a solution of **7** (0.03 mmol, 9.0 mg) in acetone (5.0 mL). Methyl iodide (0.4 mL, Sigma-Aldrich, Inc., Saint Louis, MO, USA) was added and the mixture was stirred for 4 days at room temperature. Then, the reaction mixture was neutralized with 1 M HCl. MeOH (5 mL) was added and the solvents were removed under reduced pressure. The mixture was extracted with EtOAc and evaporated to dryness to obtain a crude extract. The main product **13** (1.5 mg) was obtained from the EtOAc extract using semipreparative HPLC with a 75% acetonitrile in water supplementing with 0.08% formic acid isocratic solvent system. The NMR data (Supplementary Figures S30–S34) for **13** were similar with those of dimethylstealthin C [22,26] and was characterized as: *O1*,*O11*,*N*-trimethylstealthin C (**13**): ^1^H (DMSO-*d*_6_, 700 MHz) *δ* 14.42 (s), 10.55 (d, *J* = 5.3 Hz), 7.53 (s), 7.42 (dd, *J* = 7.9, 8.0 Hz), 7.25 (d, *J* = 8.0 Hz), 7.15 (s), 6.69 (d, *J* = 7.9 Hz), 3.96 (s, 3H), 3.75 (s, 3H), 3.60 (d, *J* = 5.3 Hz), 2.43 (s, 3H); ^13^C (DMSO-*d*_6_, 176 MHz) 177.1 (C, C-10), 163.4 (C, C-11), 162.7 (C, C-9), 155.8 (C, C-4), 143.2 C (C, C-5), 140.9 (C, C-2), 136.9 (C, C-5a), 134.8 (C, C-11a), 133.3 (CH, C-7), 124.1 (C, C-4b), 123.7 (C, C-4a), 120.8 (CH, C-1), 118.2 (CH, C-3), 118.0 (C, C-9a), 114.3 (CH, C-6), 113.9 (CH, C-8), 106.6 (C, C-10a), 63.7 (CH_3_, 5-OCH_3_), 56.3 (CH_3_, 4-OCH_3_), 33.3 (CH_3_, NCH_3_), 21.8 (CH_3_, C-12); (+)-HRESIMS *m/z* 350.1386 [M + H]^+^ (calcd for C_21_H_20_NO_4_, 350.1387, Supplementary Figure S29).

Stealthin D (**9**): yellowish needles; ${\left[\alpha \right]}_{D}^{25}$ − 2.3 (*c* 0.2, MeOH); UV (MeOH) *λ*_max_ (log ε) 205 (3.92), 242 (3.64), 343 (3.29), 408 (3.23) nm; IR *ν*_max_ 3368, 1456, 1020 cm^−1^; (+)-HRESIMS *m/z* 364.1183 [M + H]^+^ (calcd for C_21_H_18_NO_5_, 364.1179).

Stealthin E (**10**): yellowish needles; ${\left[\alpha \right]}_{D}^{25}$ + 4.1 (*c* 0.2, MeOH); (+)-HRESIMS *m/z* 378.1334 [M + H]^+^ (calcd for C_22_H_20_NO_5_, 378.1336).

Stealthin F (**11**): yellowish needles; ${\left[\alpha \right]}_{D}^{25}$ + 1.3 (*c* 0.2, MeOH); UV (MeOH) *λ*_max_ (log ε) 204 (3.88), 244 (3.66), 346 (3.40), 422 (3.27) nm; IR *ν*_max_ 3348, 1456, 1020 cm^−1^; (+)-HRESIMS *m/z* 378.1334 [M + H]^+^ (calcd for C_22_H_20_NO_5_, 378.1336).

Stealthin G (**12**): yellowish needles; ${\left[\alpha \right]}_{D}^{25}$ + 3.5 (*c* 0.2, MeOH); UV (MeOH) *λ*_max_ (log ε) 206 (3.93), 342 (3.24), 405 (3.13) nm; IR *ν*_max_ 3310, 1456, 1022 cm^−1^; (+)-HRESIMS *m/z* 378.1343 [M + H]^+^ (calcd for C_22_H_20_NO_5_, 378.1336).

### 3.5. X-ray Crystallographic Analysis

Two yellow or reddish crystals of **9** and **11** were obtained in aqueous methanol, respectively. The crystal data were collected with a Rigaku XtaLab PILATUS3 R 200K diffractometer with Cu Kα radiation (λ = 1.54184 Å). The structures were solved by direct methods (SHELXL, 2018/1) and refined using full-matrix least-squares difference Fourier techniques. Crystallographic data for **9** and **11** have been deposited in the Cambridge Crystallographic Data Center with the deposition numbers CCDC 1887925 and CCDC 1887926, respectively. A copy of the data can be obtained, free of charge, on application to the Director, CCDC,12 Union Road, Cambridge CB21EZ, UK (fax: +44(0)-1233-336033; e-mail: deposit@ccdc.cam.ac.uk).

## Figures and Tables

**Table 1:** ^1^H NMR (700 MHz) data for stealthins D–G (**9**–**12**) in DMSO-*d*_6_, *δ*_H_, multi (*J* in Hz).

No.	9	10	11	12
2	6.85, s	6.84, s	6.88, s	6.83, s
4	7.35, s	7.34, s	7.38, s	7.37, s
8	7.17, d (8.2)	7.16, dd (0.8, 8.2)	7.19, d (8.2)	7.12, d (8.2)
9	7.55, dd (7.3, 8.2)	7.55, dd (7.4, 8.2)	7.54, dd (7.4, 8.2)	7.54, dd (7.2, 8.2)
10	7.23, d (7.3)	7.24, dd (0.8, 7.4)	7.33, d, (7.4)	7.22, d (7.2)
12	2.33, s	2.32, s	2.32, s	2.32, s
13	2.87, d (13.7)	2.87, d (13.8)	3.52, q (7.0)	3.35, q (7.2)
	3.40, d (13.7)	3.31, d (13.8)		
15	1.71, s	2.02, dq (18.2, 7.2)	1.75, s	1.91, s
		2.12, dq (18.2, 7.2)		
16		0.62, t (7.2)	0.73, d (7.0)	0.65, d (7.2)
1-OH	9.69, s	9.66, s	9.69, s	9.61, s
5-NH_2_	8.12, s	8.11, s	8.23, s	8.19, s
	8.55, s	8.54, s	8.58, s	8.59, s
7-OH	13.32, s	13.32, s	13.45, s	13.17, s

**Table 2:** ^13^C NMR (176 MHz) data for stealthins D–G (**9**–**12**) in DMSO-*d*_6_ (*δ*_C_, type).

No.	9	10	11	12
1	154.3, C	154.2, C	153.7, C	154.0, C
2	119.6, CH	119.6, CH	120.3, CH	119.8, CH
3	139.7, C	139.7, C	139.9, C	140.0, C
4	114.1, CH	114.1, CH	114.4, CH	114.3, CH
4a	138.0, C	137.9, C	138.0, C	138.5, C
5	159.1, C	159.1, C	159.1, C	159.4, C
5a	105.0, C	105.2, C	103.8, C	105.6, C
6	182.7, C	182.6, C	182.6, C	182.7, C
6a	118.8, C	118.9, C	119.8, C	118.8, C
7	160.4, C	160.4, C	160.4, C	160.1, C
8	122.2, CH	122.1, CH	123.2, CH	121.6, CH
9	134.3, CH	134.2, CH	134.0, CH	134.2, CH
10	118.0, CH	118.0, CH	118.3, CH	118.3, CH
10a	136.6, C	136.7, C	135.8, C	138.2, C
11	196.6, C	196.7, C	198.3, C	198.2, C
11a	60.9, C	60.9, C	65.6, C	65.2, C
11b	127.6, C	127.8, C	127.0, C	126.2, C
12	21.1, CH_3_	21.1, CH_3_	20.9, CH_3_	21.0, CH_3_
13	46.8, CH_2_	46.3, CH_2_	52.7, CH	52.3, CH
14	204.1, C	206.5, C	208.1, C	209.5, C
15	30.9, CH_3_	36.3, CH_2_	30.7, CH_3_	29.1, CH_3_
16		7.2, CH_3_	13.3, CH_3_	12.5, CH_3_

## Supplementary Materials

The following are available online at https://www.mdpi.com/1660-3397/17/3/150/s1, Figure S1: Construction *flsN3* inactivation mutant Δ*flsN3*, Figure S2: LC-MS analysis of pyrazolofluostatin A and fluostatin R, and chiral HPLC analysis of **9**–**12**, Figure S3: UV and LC-MS analysis of the analogue related to salinipyrone A (**8**), Figure S4: HPLC traces of the methylation of stealthin C (**7**), Figure S5: HRESIMS (**a**), UV (**b**), IR (**c**) of stealthin D (**9**), Figure S6: The ^1^H NMR spectrum of stealthin D (**9**) in DMSO-*d*_6_, Figure S7: The ^13^C and DEPT 135 NMR spectra of stealthin D (**9**) in DMSO-*d*_6_, Figure S8: The ^1^H-^1^H COSY spectrum of stealthin D (**9**) in DMSO-*d*_6_, Figure S9: The HSQC spectrum of stealthin D (**9**) in DMSO-*d*_6_, Figure S10: The HMBC spectrum of stealthin D (**9**) in DMSO-*d*_6_, Figure S11: The HRESIMS spectrum of stealthin E (**10**), Figure S12: The ^1^H NMR spectrum of stealthin E (**10**) in DMSO-*d*_6_, Figure S13: The ^13^C NMR and DEPT 135 spectra of stealthin E (**10**) in DMSO-*d*_6_, Figure S14: The ^1^H-^1^H COSY spectrum of stealthin E (**10**) in DMSO-*d*_6_, Figure S15: The HSQC spectrum of stealthin E (**10**) in DMSO-*d*_6_, Figure S16: The HMBC spectrum of stealthin E (**10**) in DMSO-*d*_6_, Figure S17: HRESIMS (**a**), UV (**b**), IR (**c**) of stealthin F (**11**), Figure S18: The ^1^H NMR spectrum of stealthin F (**11**) in DMSO-*d*_6_, Figure S19: The ^13^C NMR and DEPT 135 spectra of stealthin F (**11**) in DMSO-*d*_6_, Figure S20: The ^1^H-^1^H COSY spectrum of stealthin F (**11**) in DMSO-*d*_6_, Figure S21: The HSQC spectrum of stealthin F (**11**) in DMSO-*d*_6_, Figure S22: The HMBC spectrum of stealthin F (**11**) in DMSO-*d*_6_, Figure S23: HRESIMS (**a**), UV (**b**), IR (**c**) of stealthin G (**12**), Figure S24: The ^1^H NMR spectrum of stealthin G (**12**) in DMSO-*d*_6_, Figure S25: The ^13^C NMR and DEPT 135 spectra of stealthin G (**12**) in DMSO-*d*_6_, Figure S26: The ^1^H-^1^H COSY spectrum of stealthin G (**12**) in DMSO-*d*_6_, Figure S27: The HSQC spectrum of stealthin G (**12**) in DMSO-*d*_6_, Figure S28: The HMBC spectrum of stealthin G (**12**) in DMSO-*d*_6_, Figure S29: The HRESIMS spectrum of trimethylstealthin C (**13**), Figure S30: The ^1^H NMR spectrum of trimethylstealthin C (**13**) in DMSO-*d*_6_, Figure S31: The ^13^C NMR and DEPT 135 spectra of trimethylstealthin C (**13**) in DMSO-*d*_6_, Figure S32: The ^1^H-^1^H COSY spectrum of trimethylstealthin C (**13**) in DMSO-*d*_6_, Figure S33: The HSQC spectrum of trimethylstealthin C (**13**) in DMSO-*d*_6_, Figure S34: The HMBC spectrum of trimethylstealthin C (**13**) in DMSO-*d*_6_, Table S1: Strains, plasmids and primers used in this study, Table S2: Crystal data and structure refinement for stealthin D (**9**), Table S3: Crystal data and structure refinement for stealthin F (**11**).
