# Supplementary material for: Discovery of Stealthin Derivatives and Implication of the Amidotransferase FlsN3 in the Biosynthesis of Nitrogen-Containing Fluostatins
Source: Mar Drugs. 2019 Mar 4;17(3):150. doi: 10.3390/md17030150 (PMC6470958; doi:10.3390/md17030150)
Supplement: Supplementary file 1 [file marinedrugs-17-00150-s001.pdf]

Supplementary Materials

Discovery of Stealthin Derivatives and Implication of the Amidotransferase FlsN3 in the Biosynthesis of Nitrogen-containing Fluostatins

Chunshuai Huang [1,2], Chunfang Yang [1], Zhuangjie Fang [1,2], Liping Zhang [1], Wenjun Zhang [1], Yiguang Zhu [1], Changsheng Zhang [1,2,*]

[1] CAS Key Laboratory of Tropical Marine Bio-resources and Ecology, Guangdong Key Laboratory of Marine Materia Medica, RNAM Center for Marine Microbiology, Institutions of South China Sea Ecology and Environmental Engineering, South China Sea Institute of Oceanology, Chinese Academy of Sciences, 164 West Xingang Road, Guangzhou 510301, China; 15969592400@163.com (C.H.); chunfangy@126.com (C.Y.); 991653913@qq.com (Z.F.); 461977340@qq.com (L.Z.); wzhang@scsio.ac.cn (W.Z.); zhuyiguang2003@163.com (Y.Z.)

[2] University of Chinese Academy of Sciences, 19 Yuquan Road, Beijing 100049, China

* Correspondence: czhang2006@gmail.com; Tel.: +86-20-8902-3038 (C.Z.)

Table of contents

Figure S1. Construction *flsN3* inactivation mutant Δ*flsN3* S4

Figure S2. Chiral HPLC analysis of **9**−**12** S5

Figure S3. UV and LC-MS analysis of the analogue related to salinipyrone A (**8**) S6

Figure S4. HPLC traces of the methylation of stealthin C (**7**) S7

Figure S5. HRESIMS (**a**), UV (**b**), IR (**c**) of stealthin D (**9**) S8

Figure S6. The ^{1}H NMR spectrum of stealthin D (**9**) in DMSO-d_6 .... S9

Figure S7. The ^{13}C and DEPT 135 NMR spectra of stealthin D (**9**) in DMSO-d_6 .... S10

Figure S8. The ^{1}H-^{1}H COSY spectrum of stealthin D (**9**) in DMSO-d_6 .... S11

Figure S9. The HSQC spectrum of stealthin D (**9**) in DMSO-d_6 .... S12

Figure S10. The HMBC spectrum of stealthin D (**9**) in DMSO-d_6 .... S13

Figure S11. The HRESIMS spectrum of stealthin E (**10**) S14

Figure S12. The ^{1}H NMR spectrum of stealthin E (**10**) in DMSO-d_6 .... S15

Figure S13. The ^{13}C NMR and DEPT 135 spectra of stealthin E (**10**) in DMSO-d_6 .... S16

Figure S14. The ^{1}H-^{1}H COSY spectrum of stealthin E (**10**) in DMSO-d_6 .... S17

Figure S15. The HSQC spectrum of stealthin E (**10**) in DMSO-d_6 .... S18

Figure S16. The HMBC spectrum of stealthin E (**10**) in DMSO-d_6 .... S19

Figure S17. HRESIMS (**a**), UV (**b**), IR (**c**) of stealthin F (**11**) S20

Figure S18. The ^{1}H NMR spectrum of stealthin F (**11**) in DMSO-d_6 .... S21

Figure S19. The ^{13}C NMR and DEPT 135 spectra of stealthin F (**11**) in DMSO-d_6 .... S22

Figure S20. The ^{1}H-^{1}H COSY spectrum of stealthin F (**11**) in DMSO-d_6 .... S23

Figure S21. The HSQC spectrum of stealthin F (**11**) in DMSO-d_6 .... S24

Figure S22. The HMBC spectrum of stealthin F (**11**) in DMSO-d_6 .... S25

Figure S23. HRESIMS (**a**), UV (**b**), IR (**c**) of stealthin G (**12**) S26

Figure S24. The ^{1}H NMR spectrum of stealthin G (**12**) in DMSO-d_6 .... S27

Figure S25. The ^{13}C NMR and DEPT 135 spectra of stealthin G (**12**) in DMSO-d_6 .... S28

Figure S26. The ^{1}H-^{1}H COSY spectrum of stealthin G (**12**) in DMSO-d_6 .... S29

Figure S27. The HSQC spectrum of stealthin G (**12**) in DMSO-d_6 .... S30
Figure S28. The HMBC spectrum of stealthin G (**12**) in DMSO-d_6 .... S31
Figure S29. The HRESIMS spectrum of trimethylstealthin C (**13**) S32
Figure S30. The ^{1}H NMR spectrum of trimethylstealthin C (**13**) in DMSO-d_6 .... S33
Figure S31. The ^{13}C NMR and DEPT 135 spectra of trimethylstealthin C (**13**) in DMSO-d_6 .. S34
Figure S32. The ^{1}H-^{1}H COSY spectrum of trimethylstealthin C (**13**) in DMSO-d_6 .... S35
Figure S33. The HSQC spectrum of trimethylstealthin C (**13**) in DMSO-d_6 .... S36
Figure S34. The HMBC spectrum of trimethylstealthin C (**13**) in DMSO-d_6 .... S37
Table S1. Strains, plasmids and primers used in this study S38
Table S2. Crystal data and structure refinement for stealthin D (**9**) S39
Table S3. Crystal data and structure refinement for stealthin F (**11**) S40
References S41

Figure S1. Construction *flsN3* inactivation mutant Δ*flsN3*

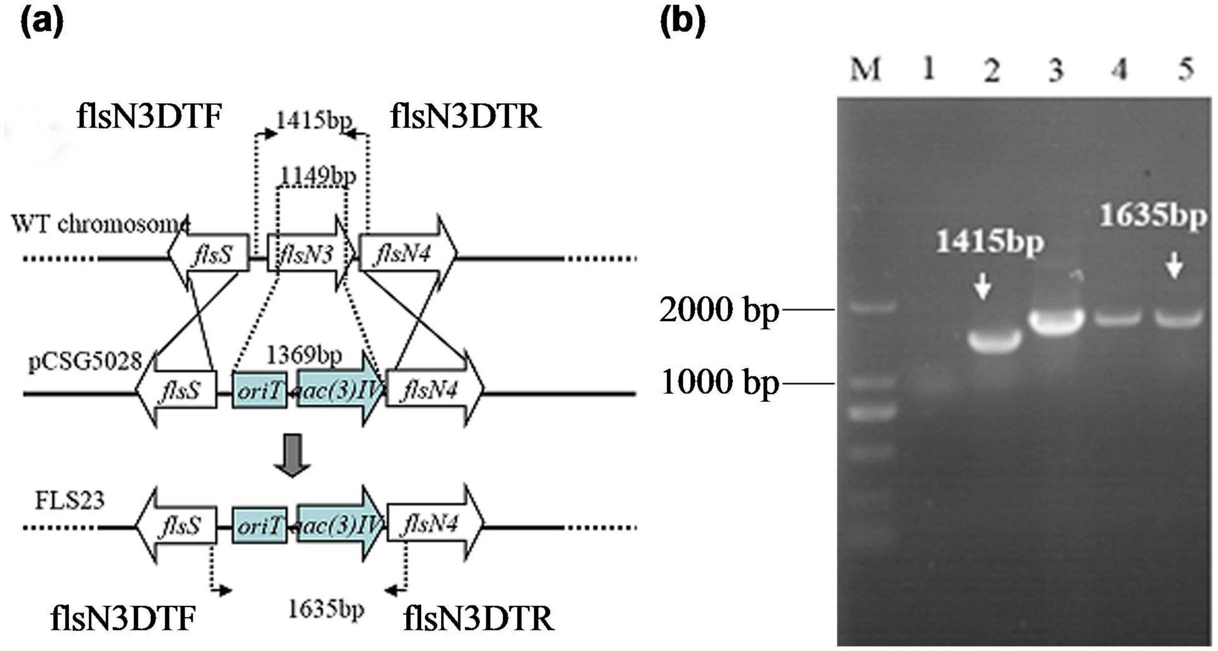

(a) Description of *flsN3* inactivation. FLS23 was constructed by replacing a 1149 bp internal *flsN3* fragment with a 1369 bp DNA fragment containing *oriT* and *acc3(IV)* in pCSG5028, resulting from a double cross-over recombination event. The location of the diagnostic PCR primers were indicated. Sizes of PCR products were also indicated: 1415 bp for the wild type strain *M. rosaria* SCSIO N160 and 1635 bp for the mutant FLS23.

(b) Gel electrophoresis of PCR products. DNA templates were from: ddH_2O (negative control, lane 1), pCSG5001 (negative control, lane 2), pCSG5017 (positive control, lane 3), Δ*flsN3* clone #1 (lane 4), Δ*flsN3* clone #2 (lane 5) and DNA marker D2000 (GenStar, lane M).

.

Figure S2. Chiral HPLC analysis of **9−12**

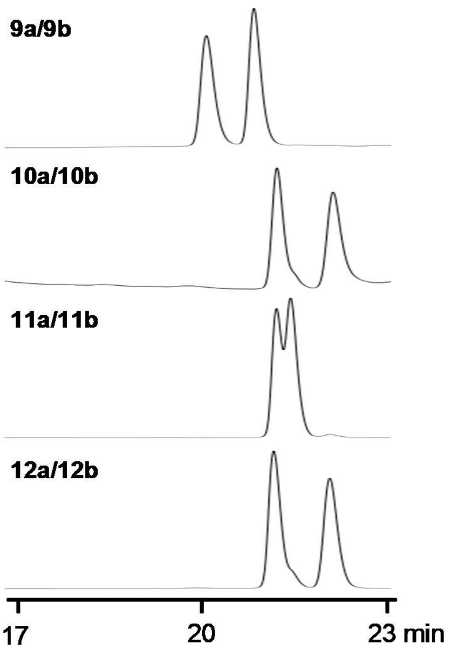

The chiral HPLC analysis was performed on an Agilent 1260 Infinity series instrument with a Chiral ND 5u (4.6 × 250 mm) chiral column (Phenomenex, Washington, CD, USA). The elution process runs the following program: 5% B to 80% B (linear gradient, 0–20 min), 80% B to 100% B (20–21 min), 100% B (isocratic elution, 21–24 min), 100% B to 5% B (24–25 min), 5% B (isocratic elution, 25–30 min). The solvent system comprises solvent A (10% acetonitrile in water supplemented with 0.08% formic acid) and B (90% acetonitrile in water). The monitoring wavelength at 430 nm.

Figure S3. UV and LC-MS analysis of the analogue related to salinipyrone A (**8**)

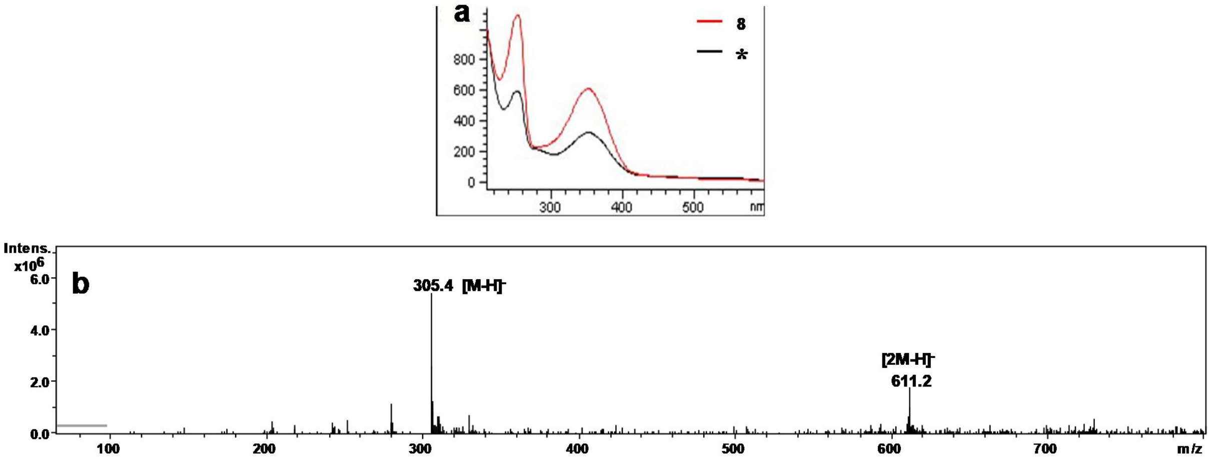

a UV comparison of salinipyrone A (**8**) with the product with the symbol "*"; **b** Negative mode ESI-MS data for the product with the symbol "*".

Figure S4. HPLC traces of the methylation of stealthin C (**7**)

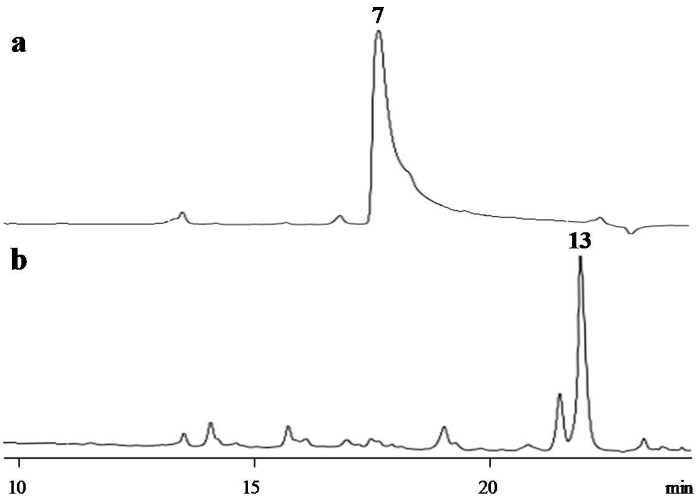

a Stealthin C (**7**) standard; **b** **7** was treated with methyl iodide.

Figure S5. HRESIMS (**a**), UV (**b**), IR (**c**) of stealthin D (**9**)

(**a**). **HR-ESI-MS**

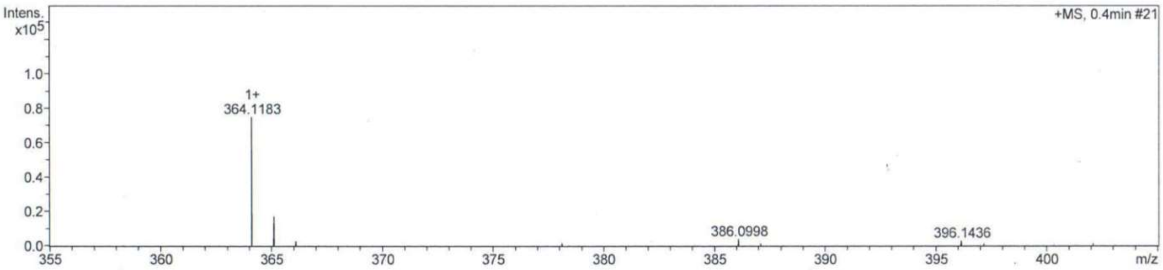

Meas. m/z	#	Ion Formula	Score	m/z	err [ppm]	err [mDa]	mSigma	rdb	e⁻ Conf	N-Rule
364.118291	1	C21H18NO5	100.00	364.117949	0.9	0.3	1.2	13.5	even	ok
386.099771	1	C21H17NNaO5	100.00	386.099893	0.3	0.1	16.0	13.5	even	ok
396.143566	1	C22H22NO6	100.00	396.144164	-1.5	-0.6	22.2	12.5	even	ok

(**b**). **UV**

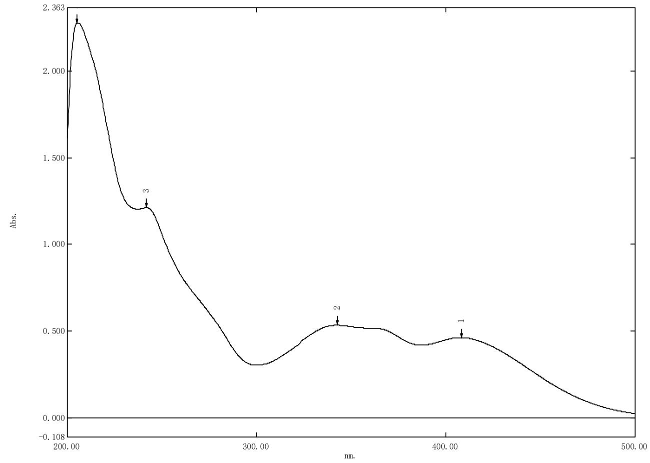

(**c**). **IR**

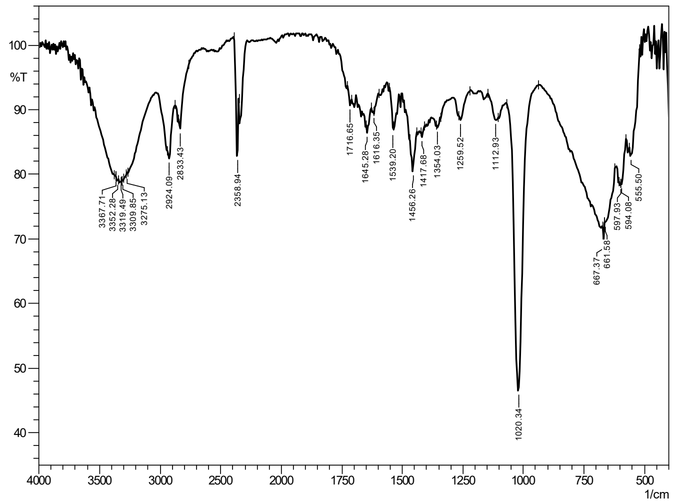

Figure S6. The ^{1}H NMR spectrum of stealthin D (**9**) in DMSO-d_6

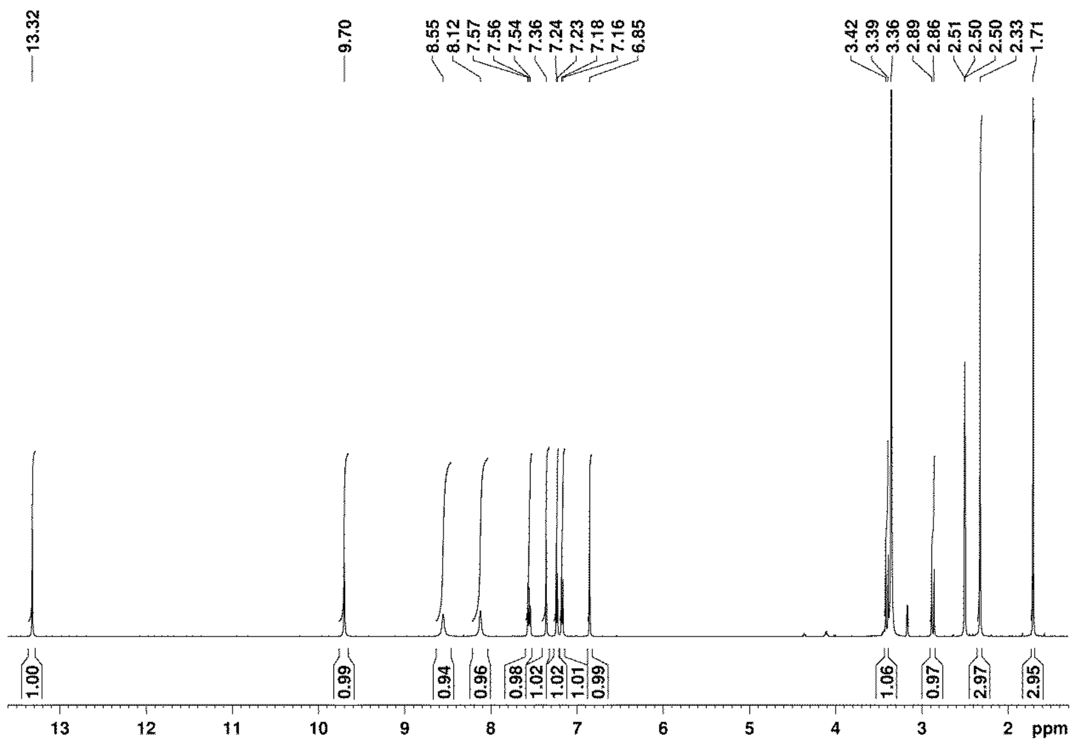

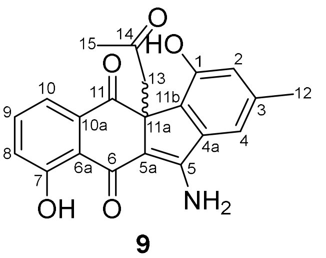

9

Figure S7. The ^{13}C and DEPT 135 NMR spectra of stealthin D (**9**) in DMSO-d_6

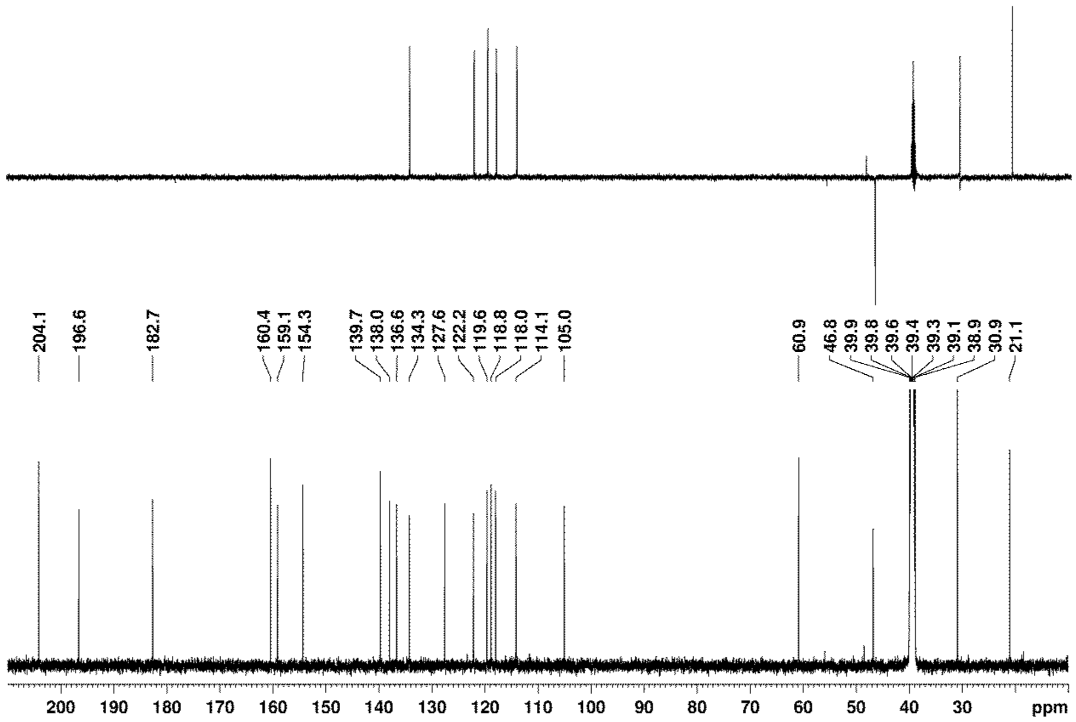

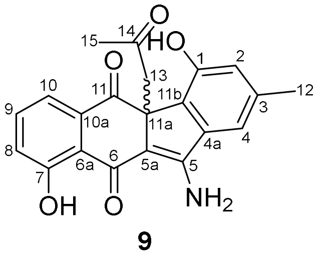

9

Figure S8. The ^1H-^1H COSY spectrum of stealthin D (**9**) in DMSO-d_6

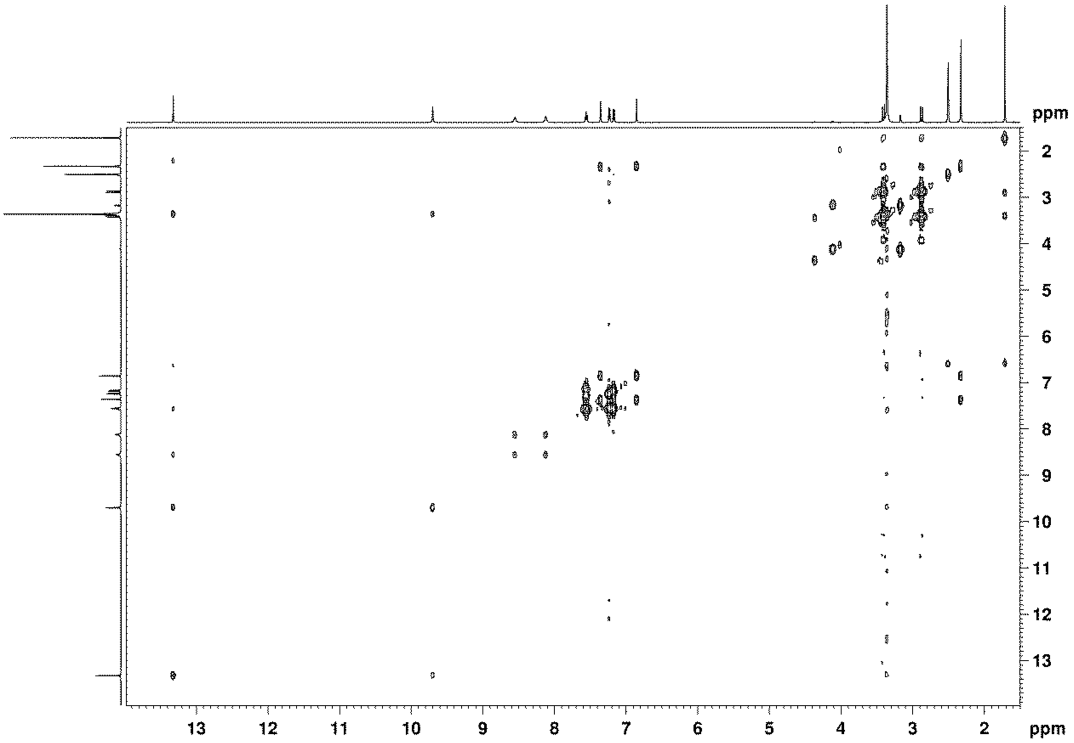

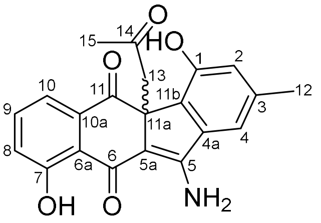

9

Figure S9. The HSQC spectrum of stealthin D (**9**) in DMSO-d_6

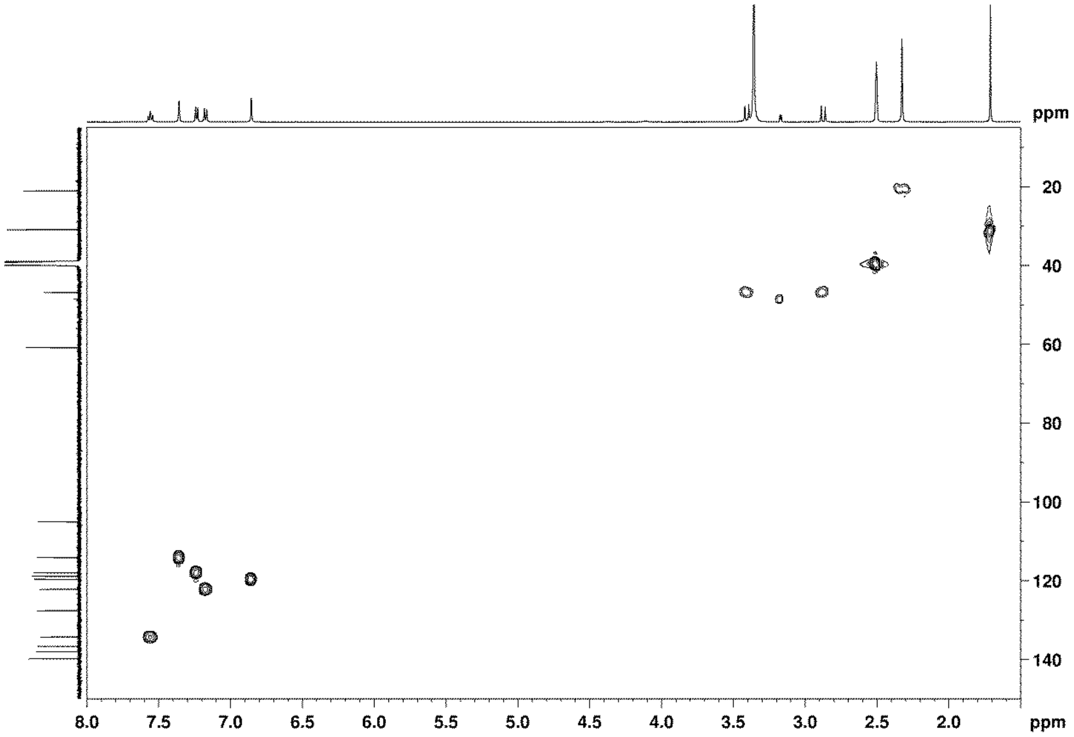

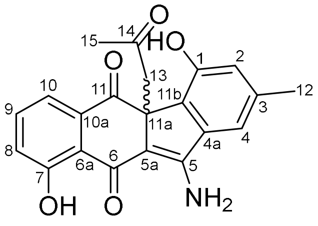

9

Figure S10. The HMBC spectrum of stealthin D (**9**) in DMSO-d_6

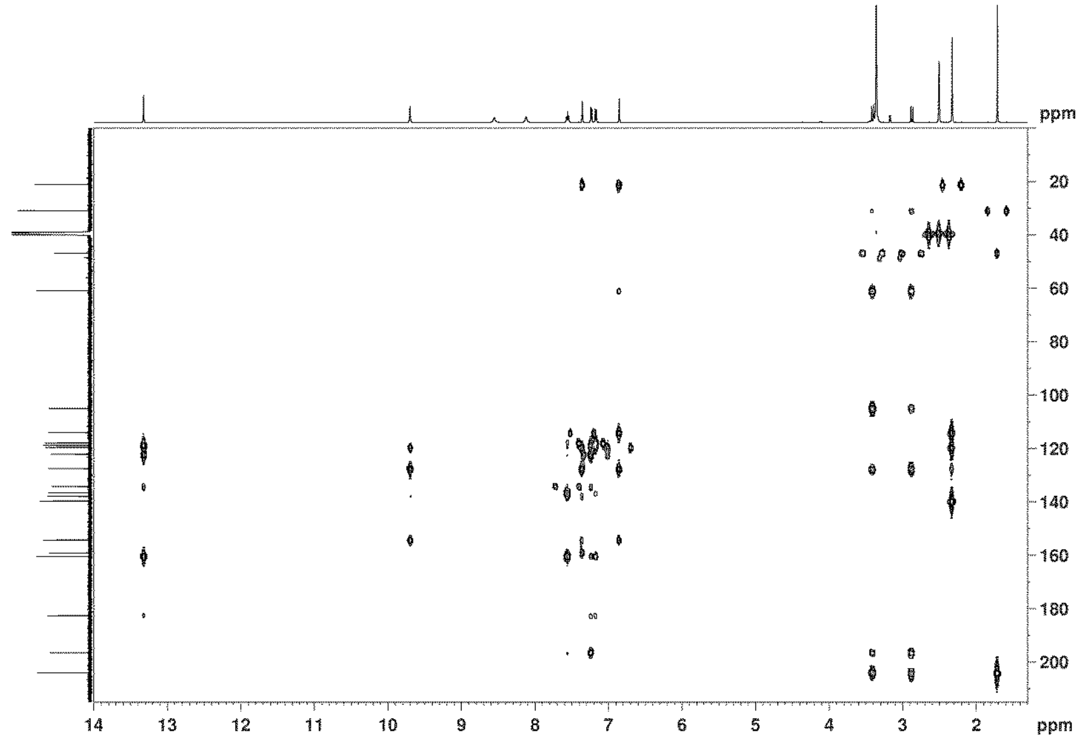

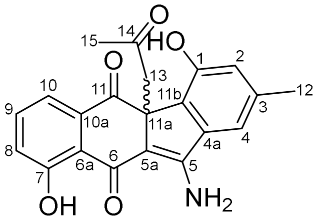

9

Figure S11. The HRESIMS spectrum of stealthin E (**10**)

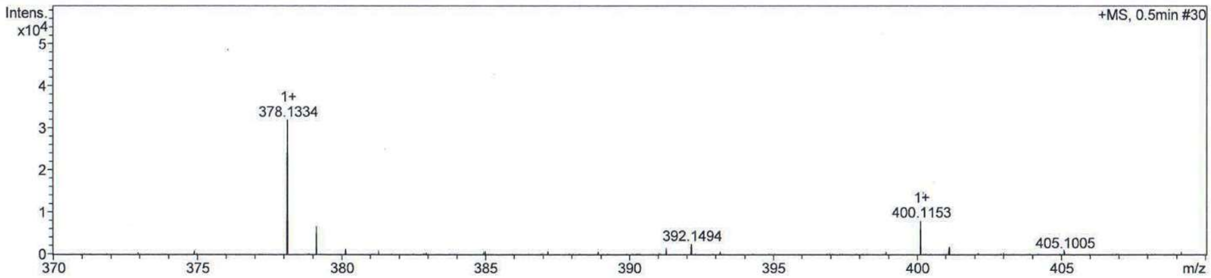

Meas. m/z	#	Ion Formula	Score	m/z	err [ppm]	err [mDa]	mSigma	rdb	e⁻ Conf	N-Rule
378.133362	1	C22H20NO5	100.00	378.133599	-0.6	-0.2	18.5	13.5	even	ok
	2	C23H16N5O	24.31	378.134937	-4.2	-1.6	35.0	18.5	even	ok
400.115293	1	C22H19NNaO5	100.00	400.115543	-0.6	-0.3	5.7	13.5	even	ok
	2	C23H15N5NaO	36.37	400.116881	-4.0	-1.6	19.5	18.5	even	ok
777.240142	1	C40H34N8NaO8	85.01	777.239181	1.2	1.0	65.6	27.5	even	ok
	2	C44H38N2NaO10	48.87	777.241866	-2.2	-1.7	67.5	26.5	even	ok

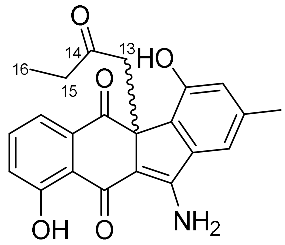

10

Figure S12. The ^{1}H NMR spectrum of stealthin E (**10**) in DMSO-d_6

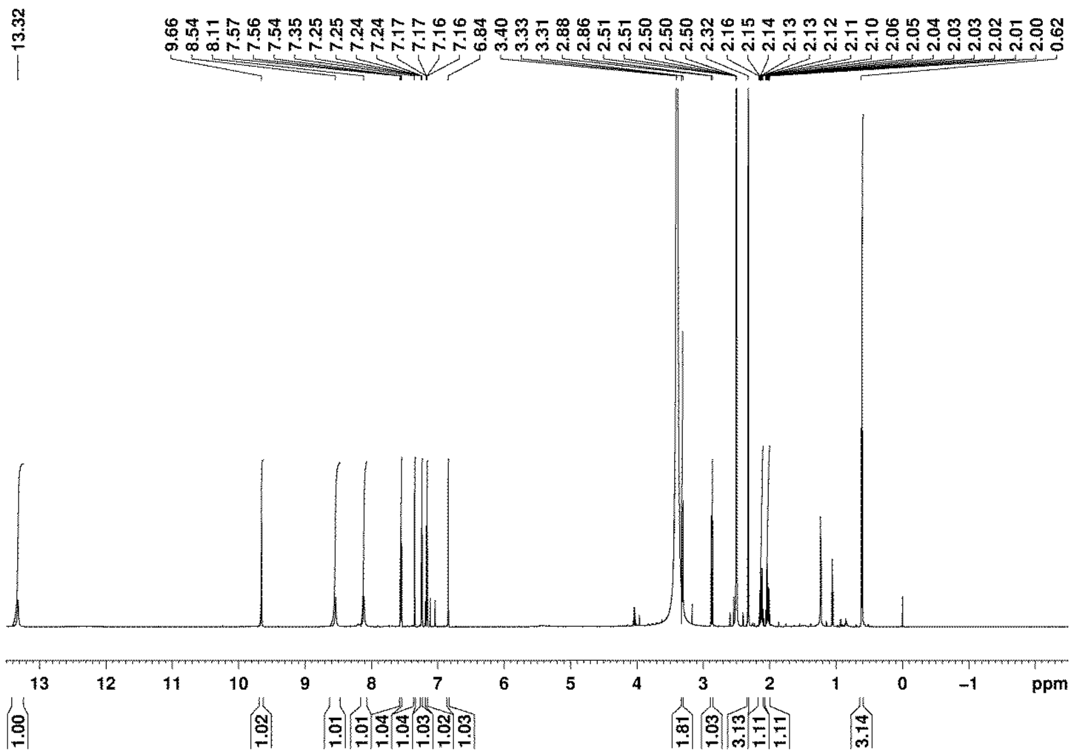

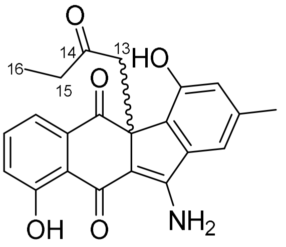

10

Figure S13. The ^{13}C NMR and DEPT 135 spectra of stealthin E (**10**) in DMSO-d_6

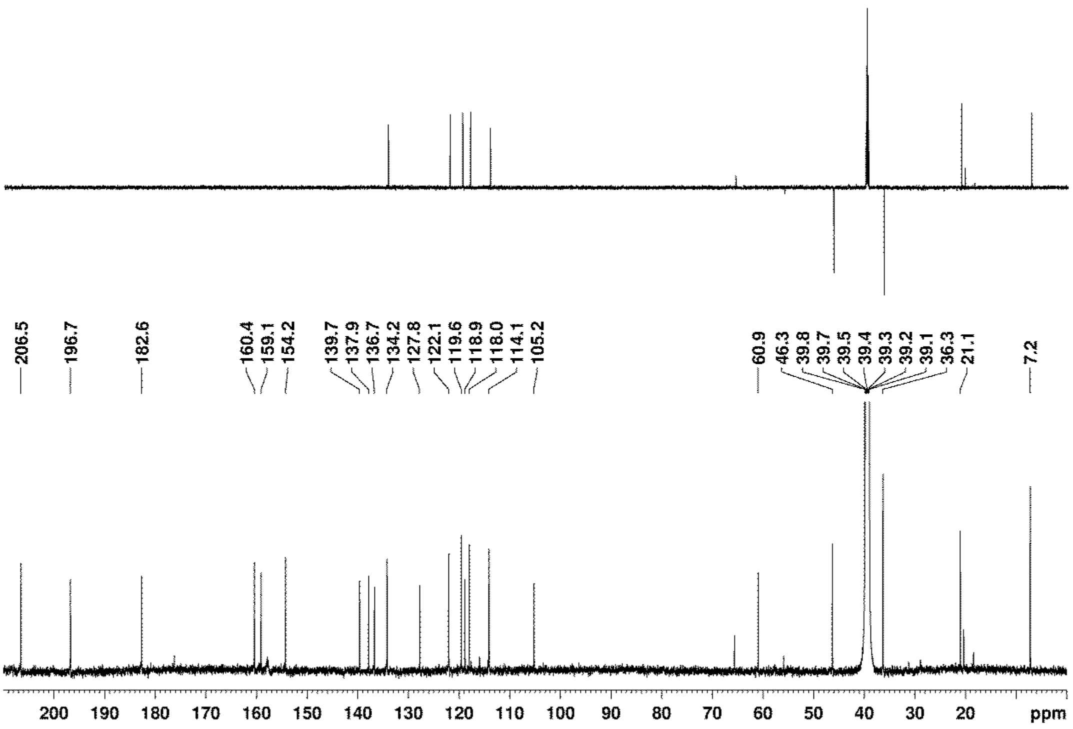

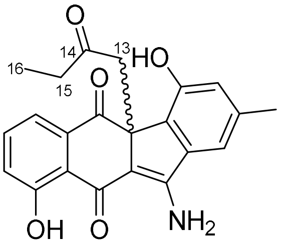

10

Figure S14. The ^{1}H-^{1}H COSY spectrum of stealthin E (**10**) in DMSO-d_6

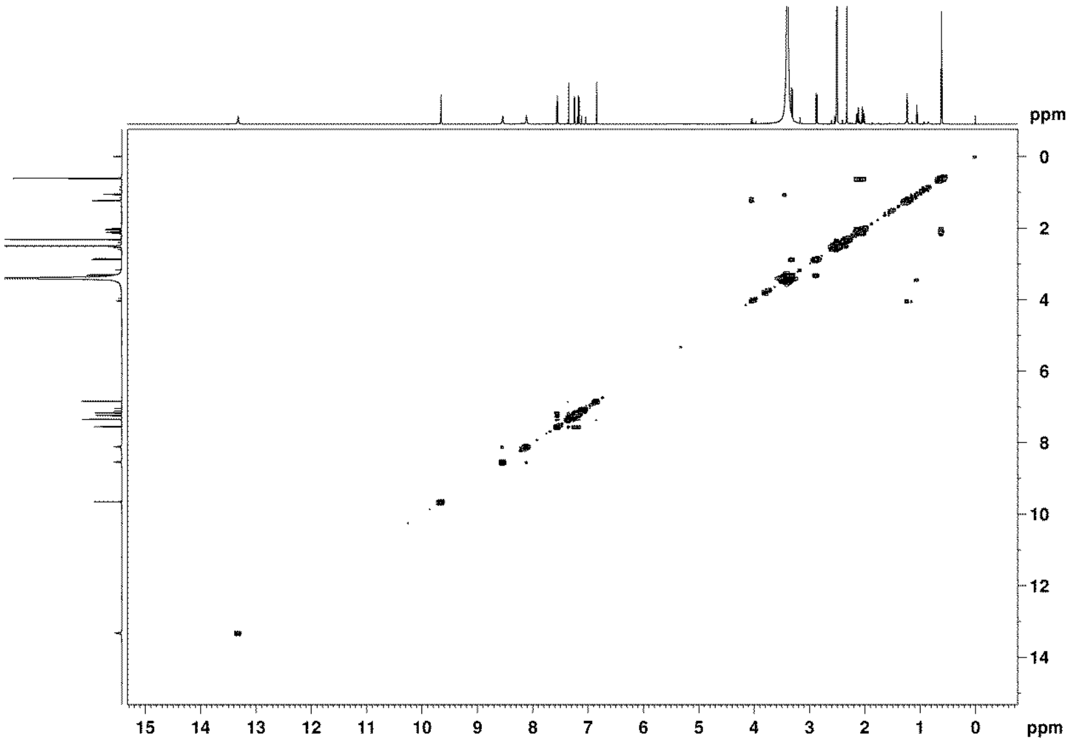

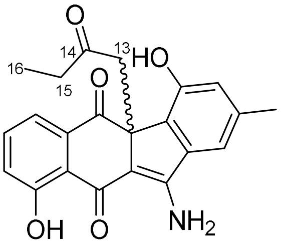

10

Figure S15. The HSQC spectrum of stealthin E (**10**) in DMSO-d_6

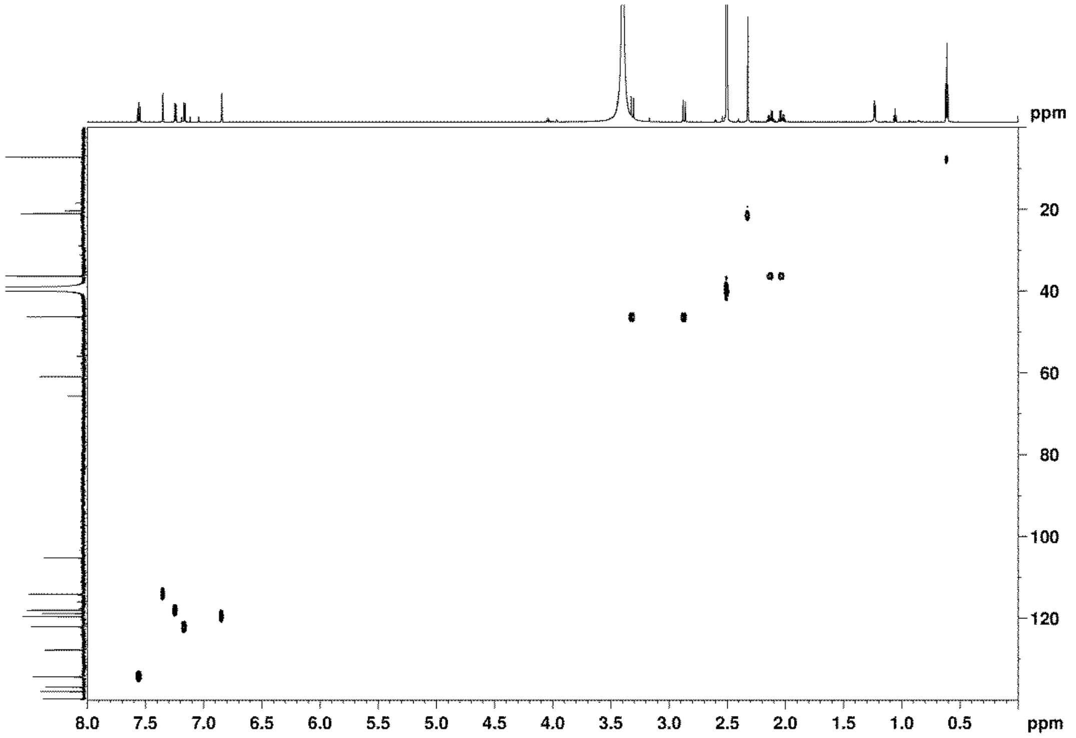

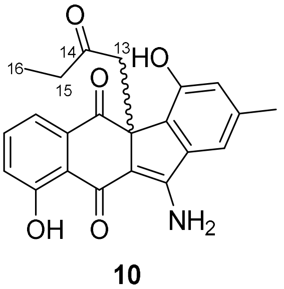

10

Figure S16. The HMBC spectrum of stealthin E (**10**) in DMSO-d_6

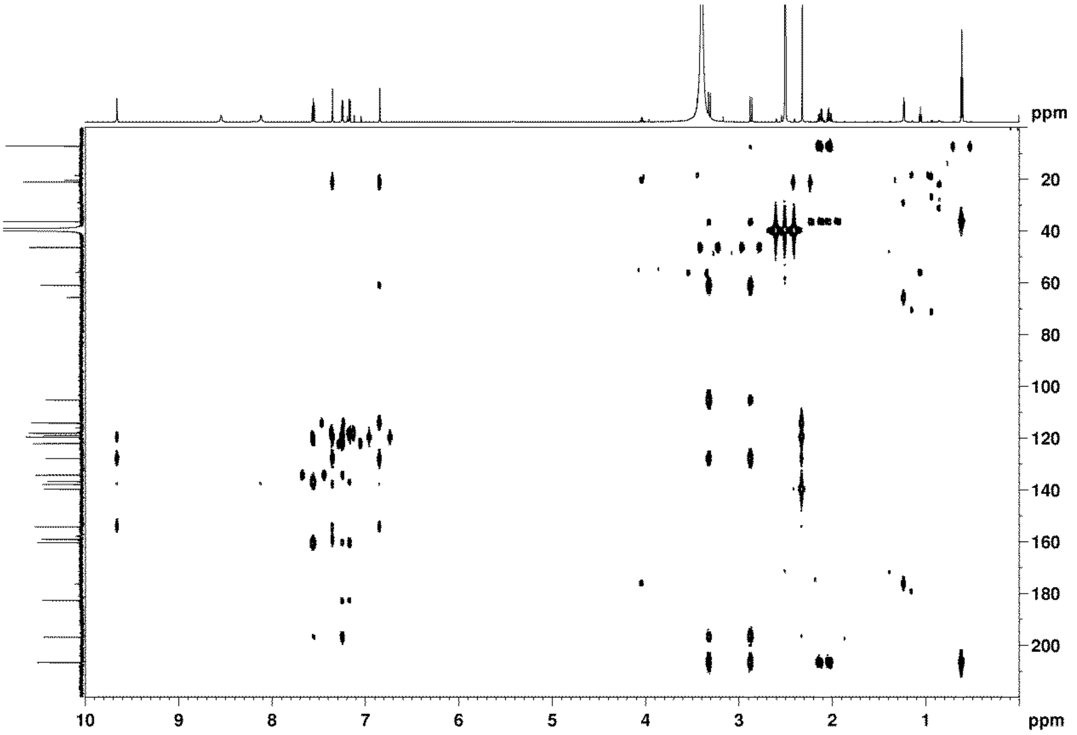

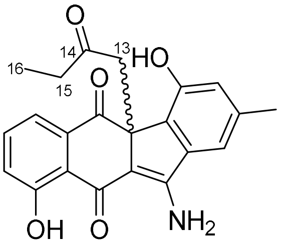

10

Figure S17. HRESIMS (**a**), UV (**b**), IR (**c**) of stealthin F (**11**)

(**a**). **HR-ESI-MS**

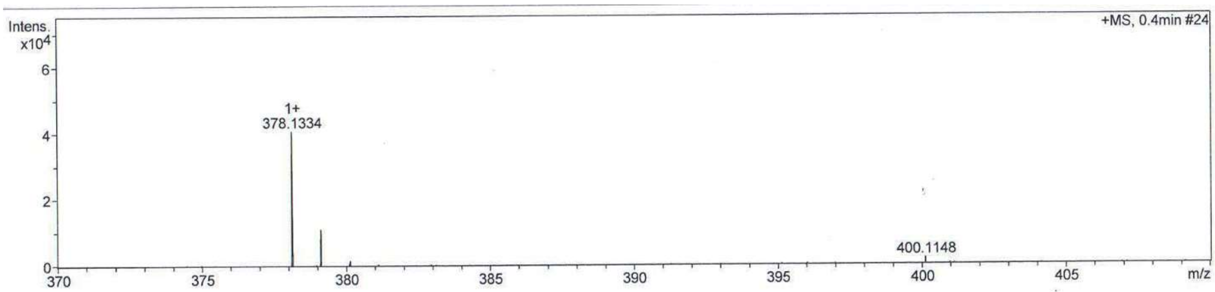

Meas. m/z	#	Ion Formula	Score	m/z	err [ppm]	err [mDa]	mSigma	rdb	e⁻ Conf	N-Rule
378.133429	1	C22H20NO5	100.00	378.133599	-0.5	-0.2	13.1	13.5	even	ok
400.114831	1	C22H19NNaO5	100.00	400.115543	1.8	0.7	38.7	13.5	even	ok
755.258947	1	C44H39N2O10	100.00	755.259922	1.3	1.0	18.7	26.5	even	ok

(**b**). **UV**

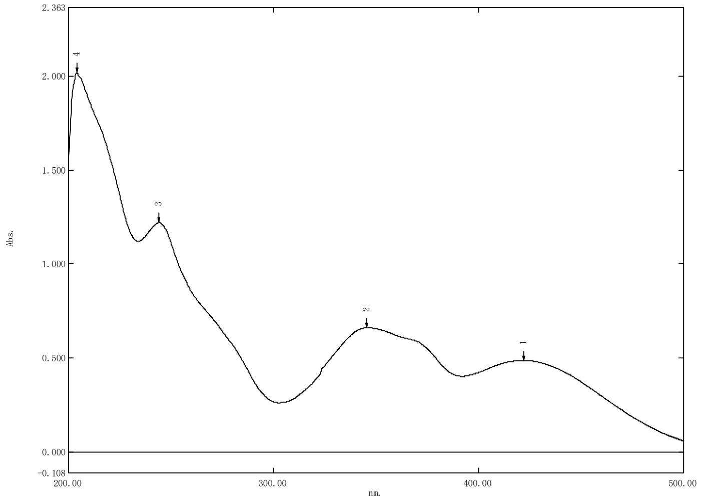

(**c**). **IR**

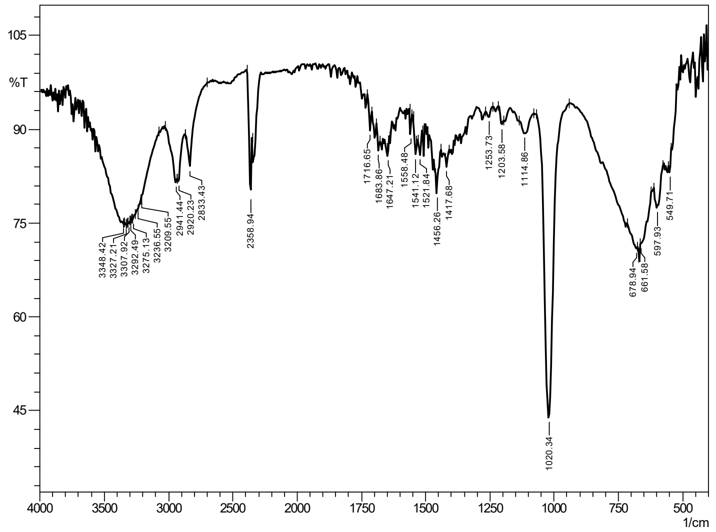

Figure S18. The ^{1}H NMR spectrum of stealthin F (**11**) in DMSO-d_6

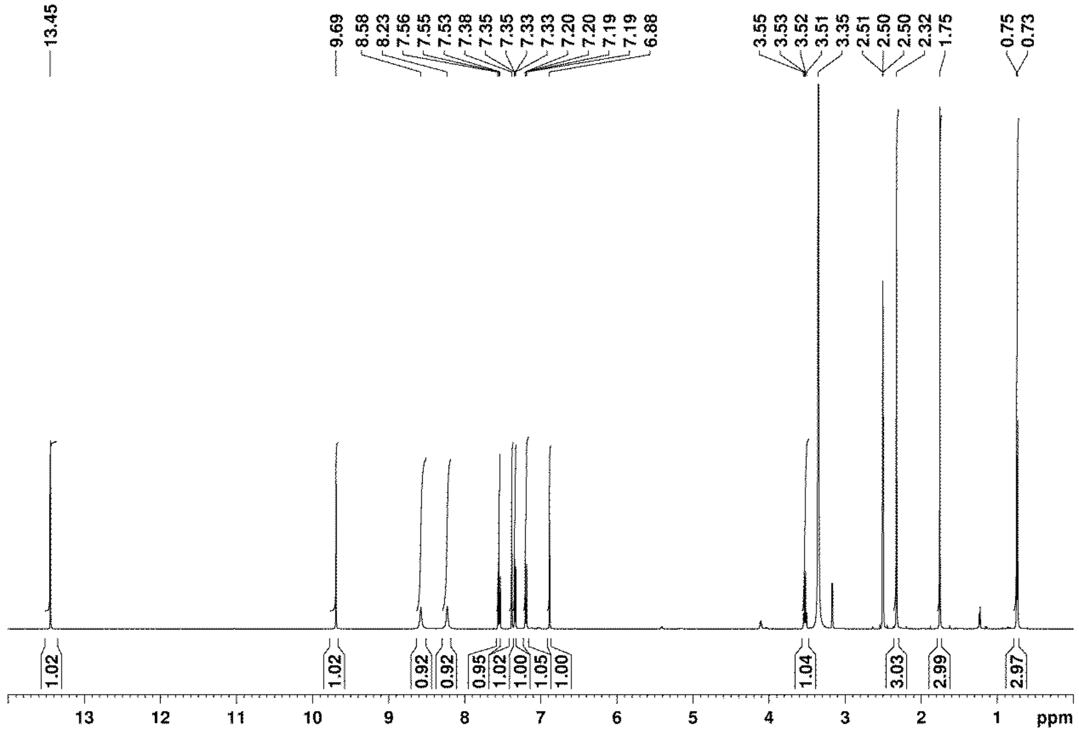

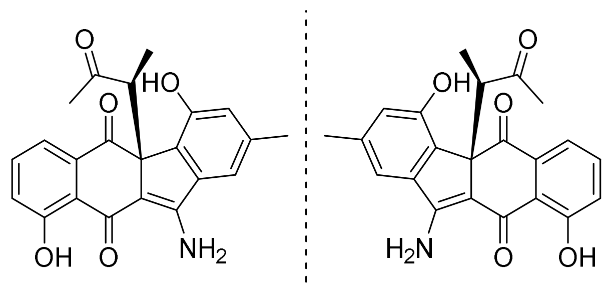

11a/11b

Figure S19. The ^{13}C NMR and DEPT 135 spectra of stealthin F (**11**) in DMSO-d_6

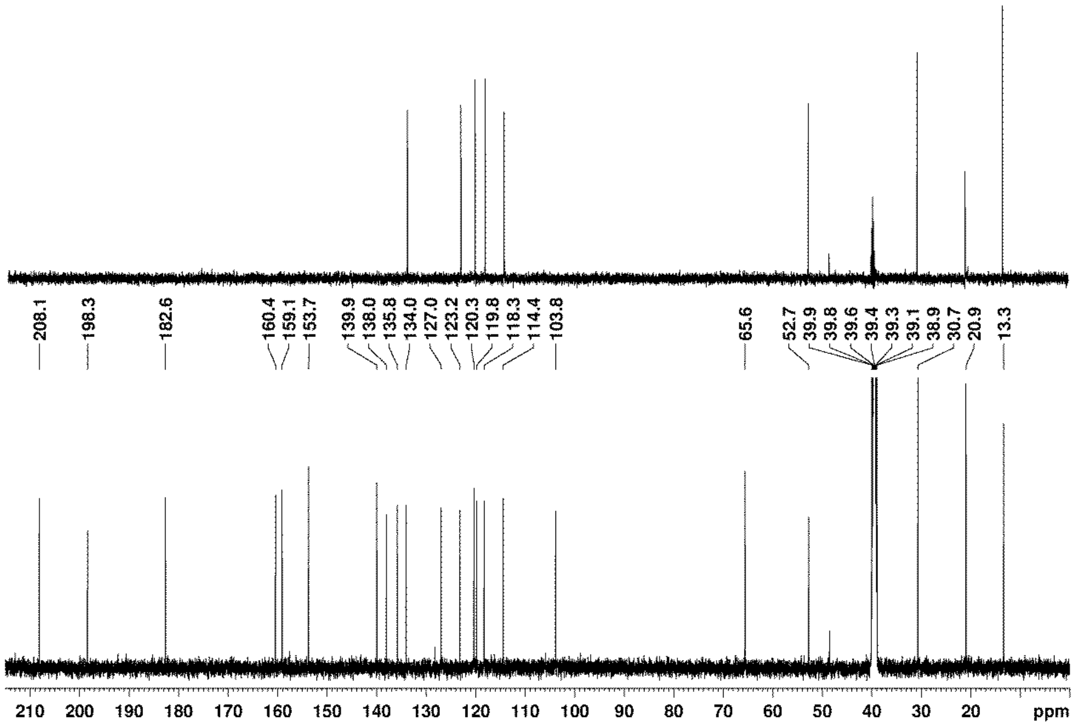

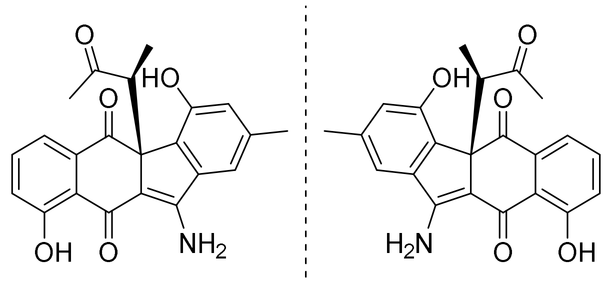

11a/11b

Figure S20. The ^{1}H-^{1}H COSY spectrum of stealthin F (**11**) in DMSO-d_6

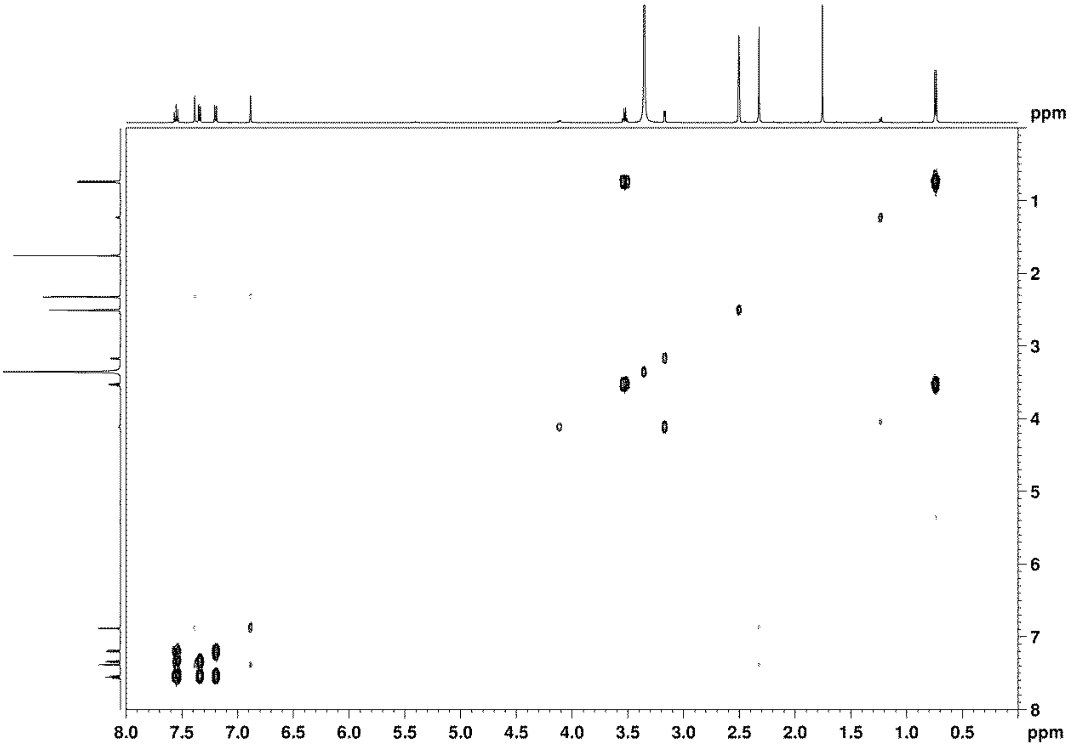

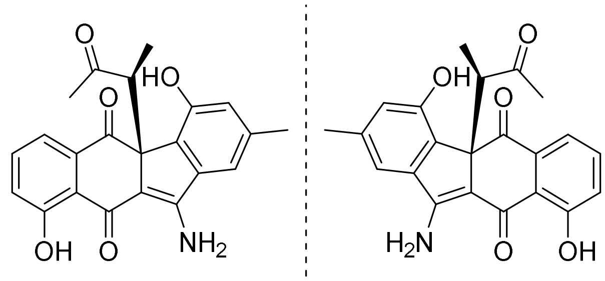

11a/11b

Figure S21. The HSQC spectrum of stealthin F (**11**) in DMSO-d_6

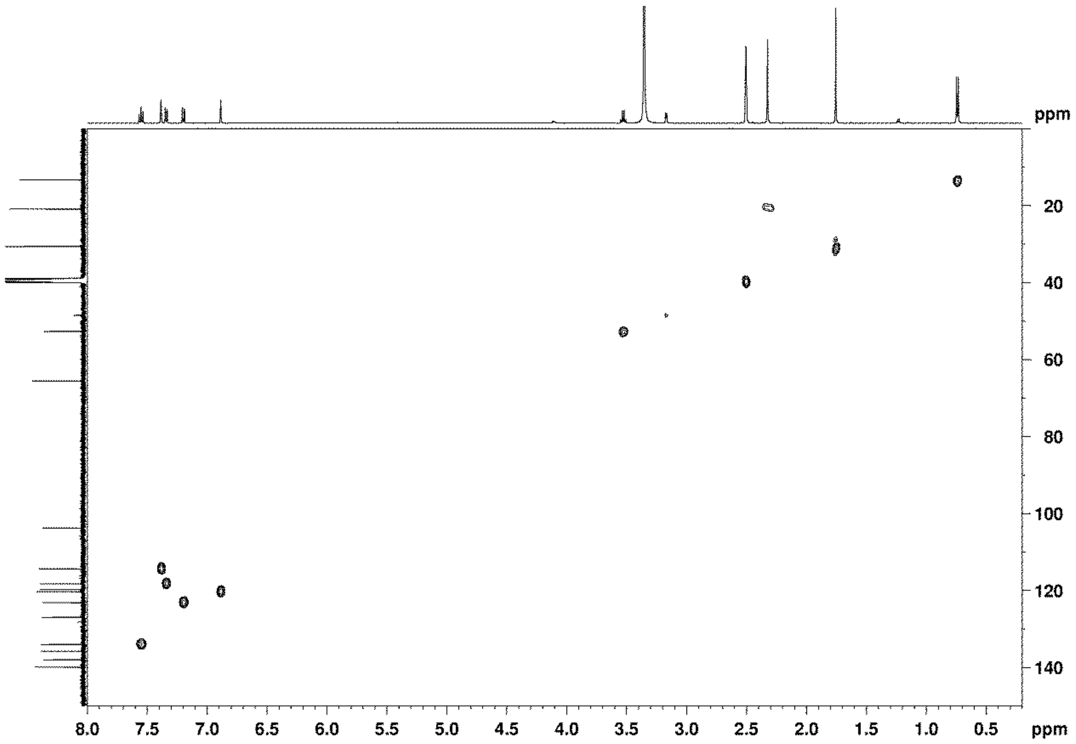

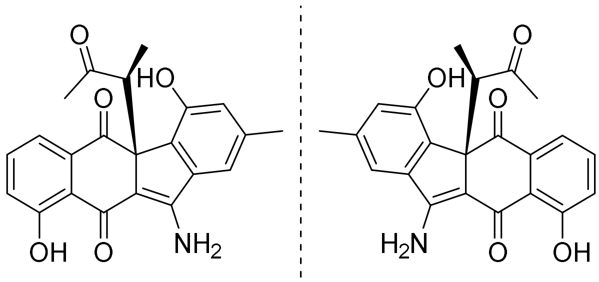

11a/11b

Figure S22. The HMBC spectrum of stealthin F (**11**) in DMSO-d_6

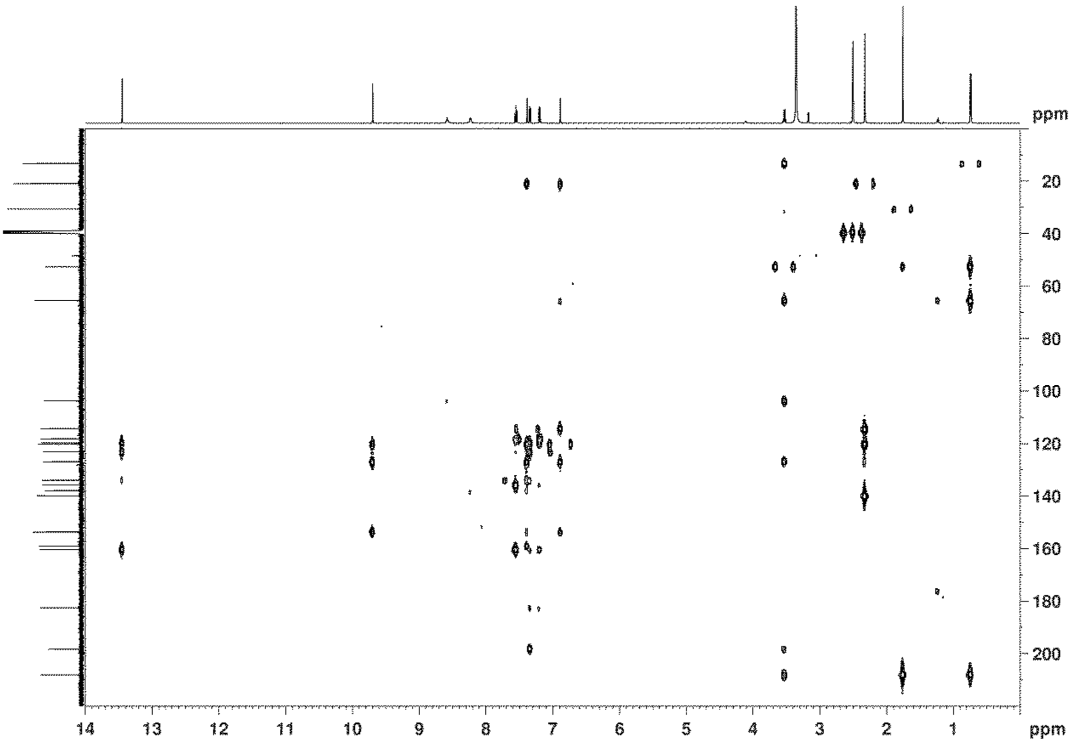

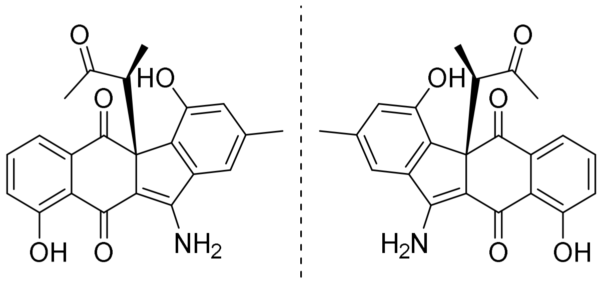

11a/11b

Figure S23. HRESIMS (**a**), UV (**b**), IR (**c**) of stealthin G (**12**)

(**a**). **HR-ESI-MS**

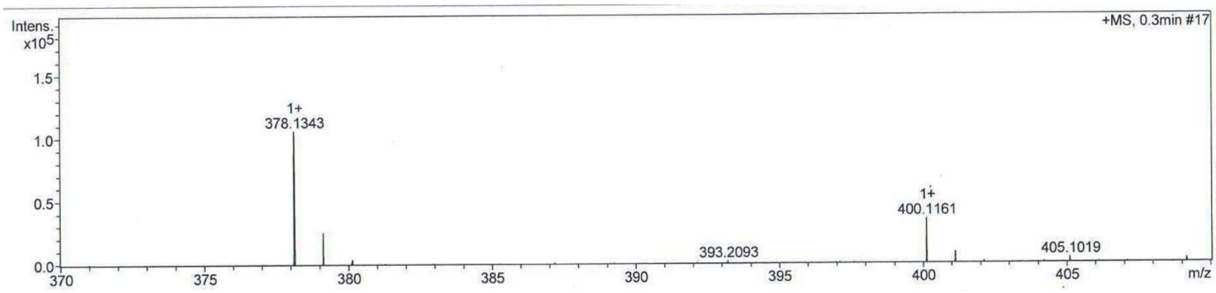

Meas. m/z	#	Ion Formula	Score	m/z	err [ppm]	err [mDa]	mSigma	rdb	e⁻ Conf	N-Rule
378.134279	1	$C_{22}H_{20}NO_5$	100.00	378.133599	-1.8	-0.7	0.4	13.5	even	ok
	2	$C_{23}H_{16}N_5O$	59.55	378.134937	-1.7	-0.7	13.9	18.5	even	ok

(**b**). **UV**

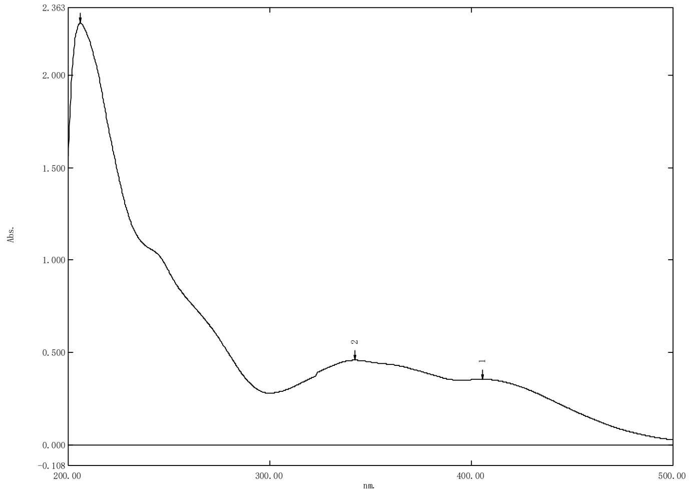

(**c**). **IR**

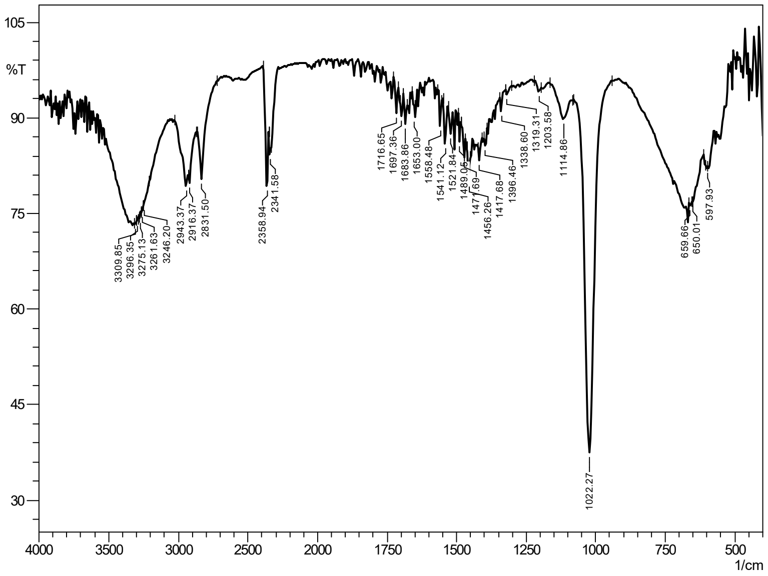

Figure S24. The ^{1}H NMR spectrum of stealthin G (**12**) in DMSO-d_6

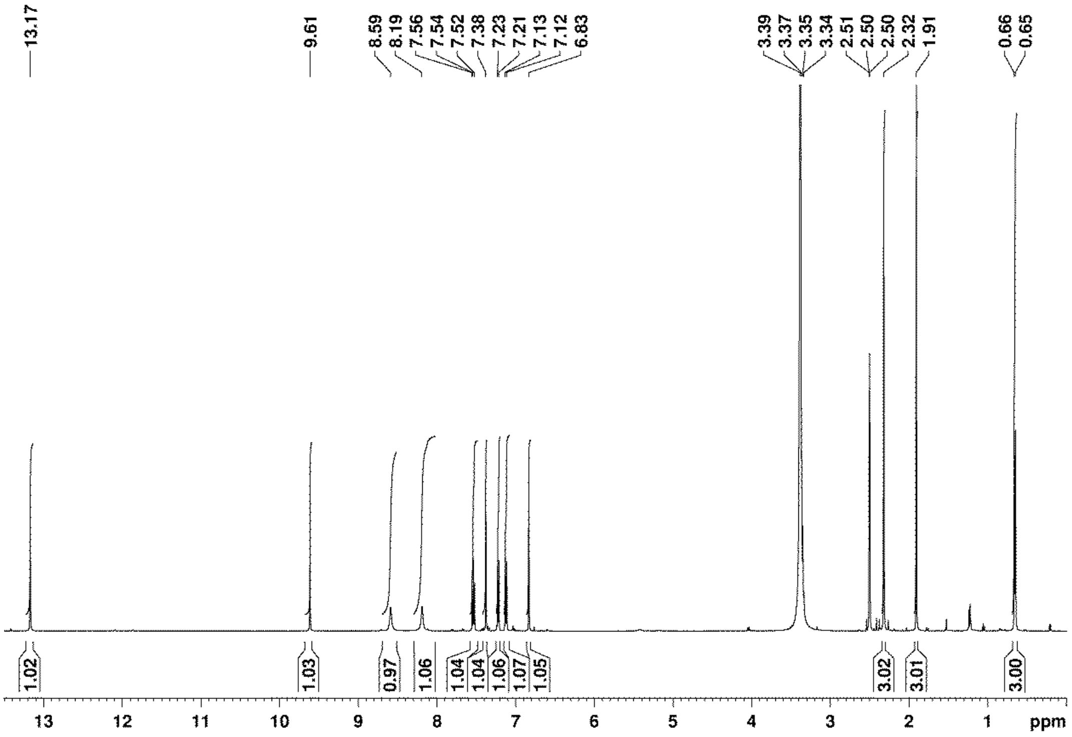

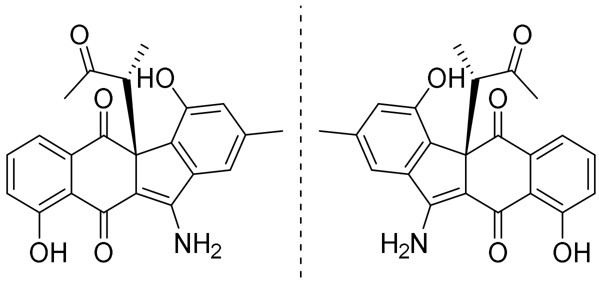

12a/12b

Figure S25. The ^{13}C NMR and DEPT 135 spectra of stealthin G (**12**) in DMSO-d_6

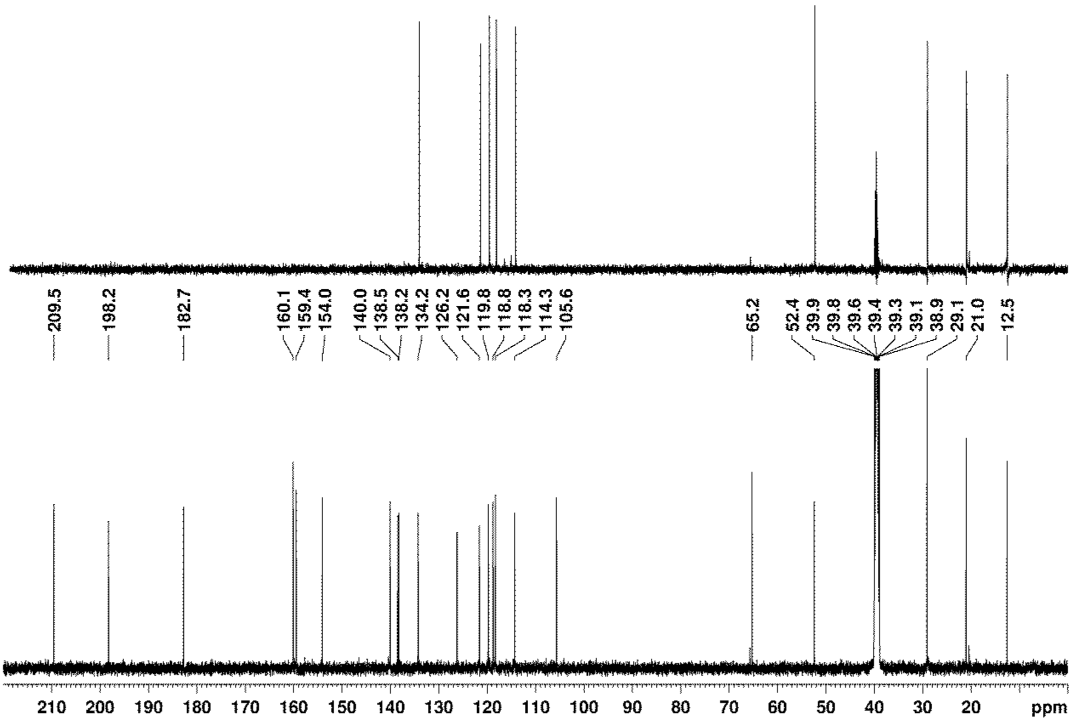

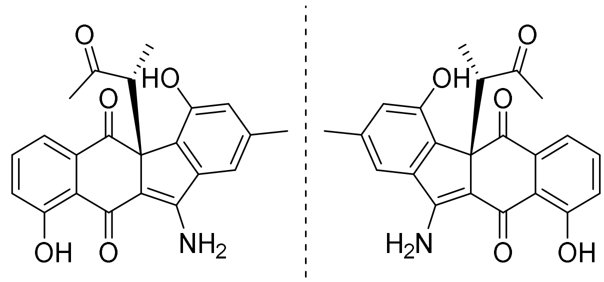

12a/12b

Figure S26. The ^{1}H-^{1}H COSY spectrum of stealthin G (**12**) in DMSO-d_6

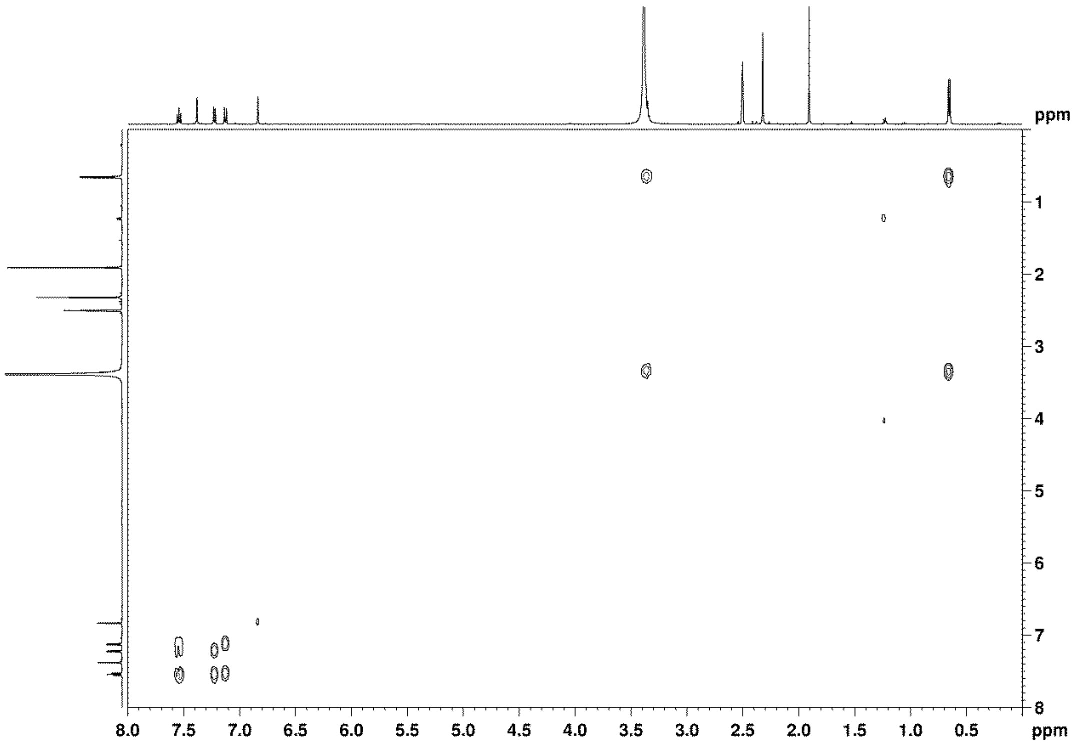

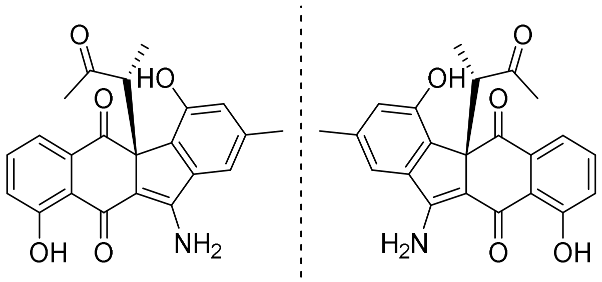

12a/12b

Figure S27. The HSQC spectrum of stealthin G (**12**) in DMSO-d_6

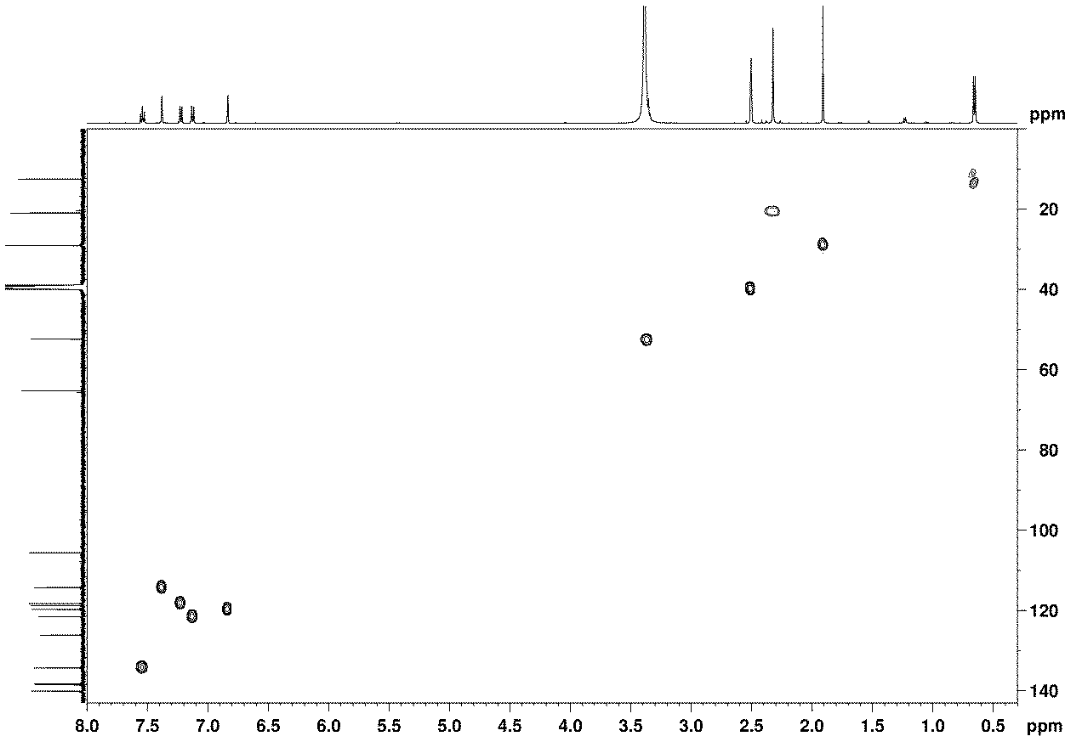

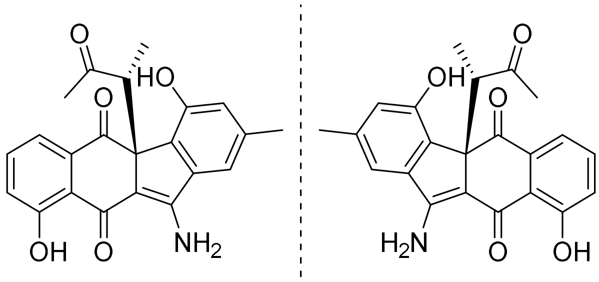

12a/12b

Figure S28. The HMBC spectrum of stealthin G (**12**) in DMSO-d_6

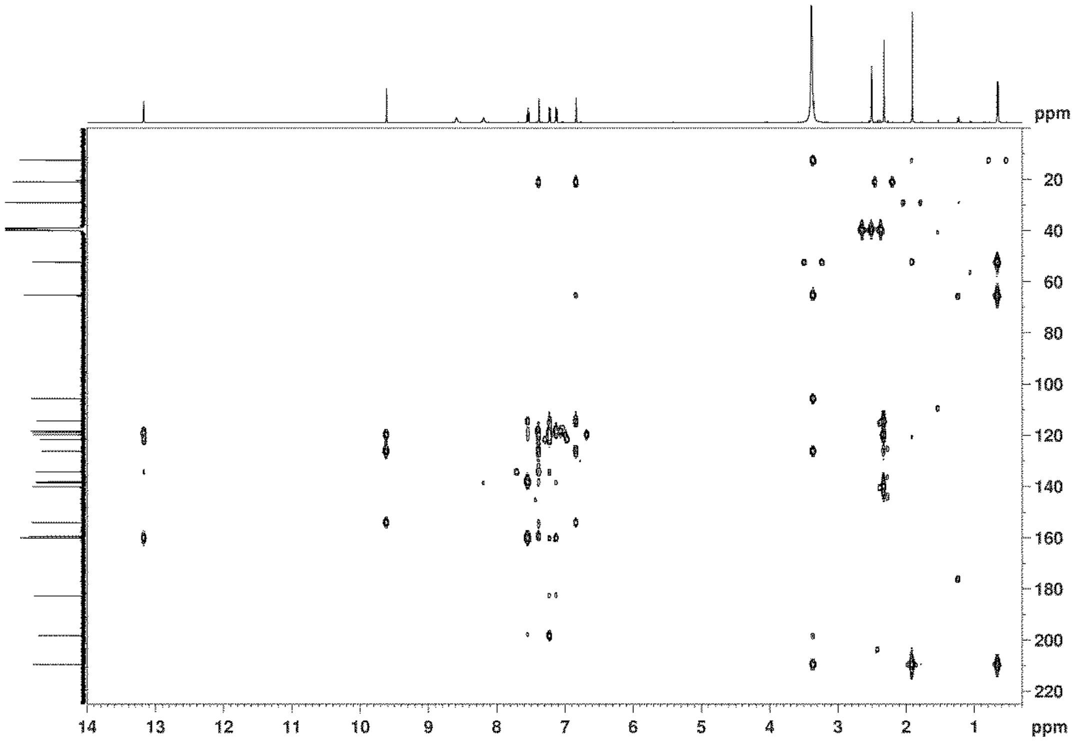

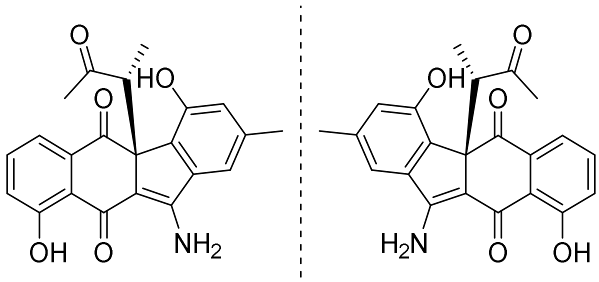

12a/12b

Figure S29. The HRESIMS spectrum of trimethylstealthin C (**13**)

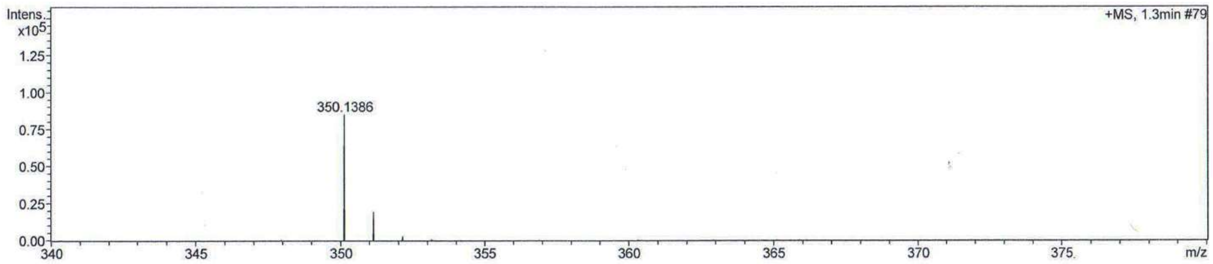

Meas. m/z	#	Ion Formula	Score	m/z	err [ppm]	err [mDa]	mSigma	rdb	e⁻ Conf	N-Rule
350.1386	1	C21H20NO4	100.00	350.1387	-0.1	-0.0	3.3	12.5	even	ok
699.2699	1	C42H39N2O8	100.00	699.2701	-0.2	-0.1	26.2	24.5	even	ok

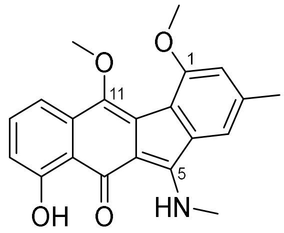

trimethylstealthin C (**13**)

Figure S30. The ^{1}H NMR spectrum of trimethylstealthin C (**13**) in DMSO-d_6

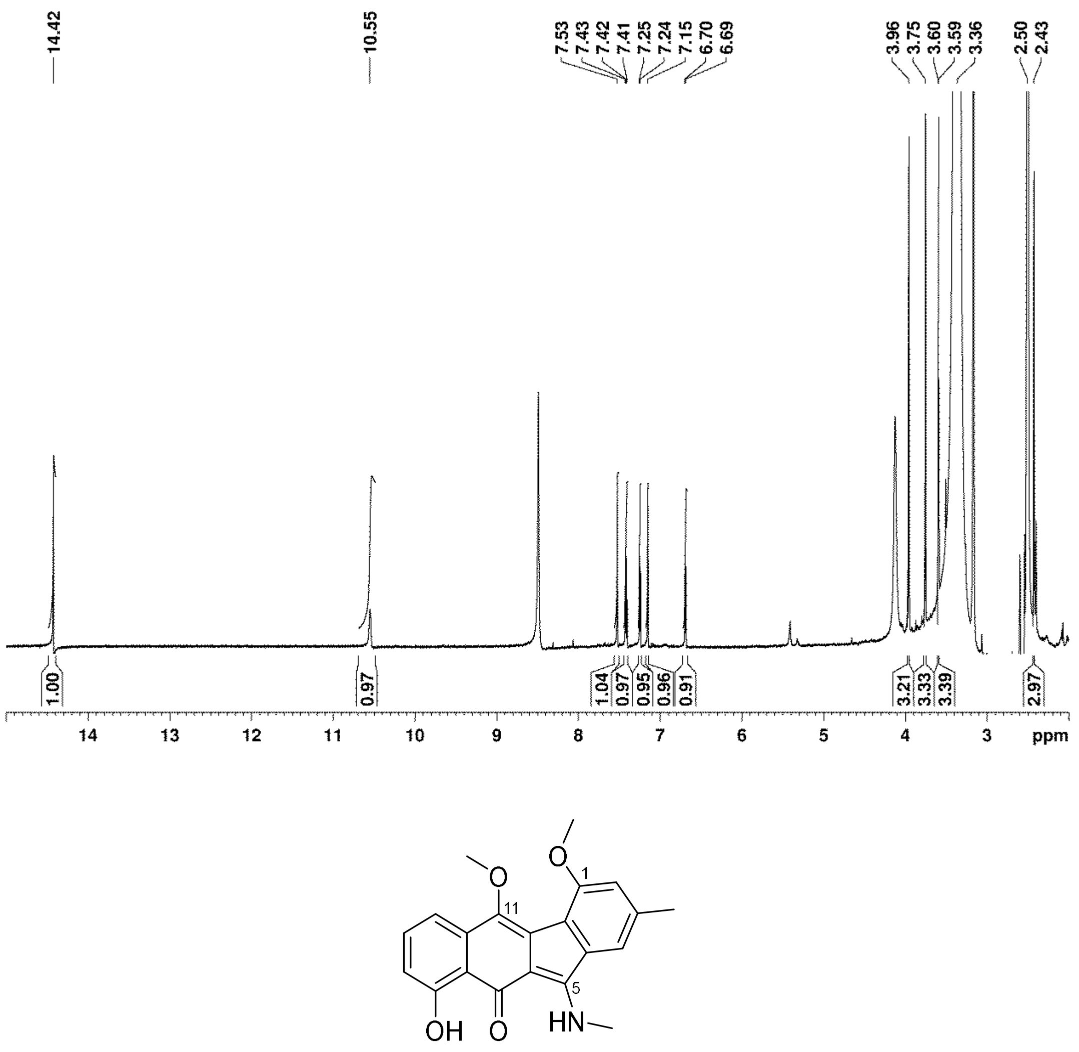

trimethylstealthin C (**13**)

Figure S31. The ^{13}C NMR and DEPT 135 spectra of trimethylstealthin C (**13**) in DMSO-d_6

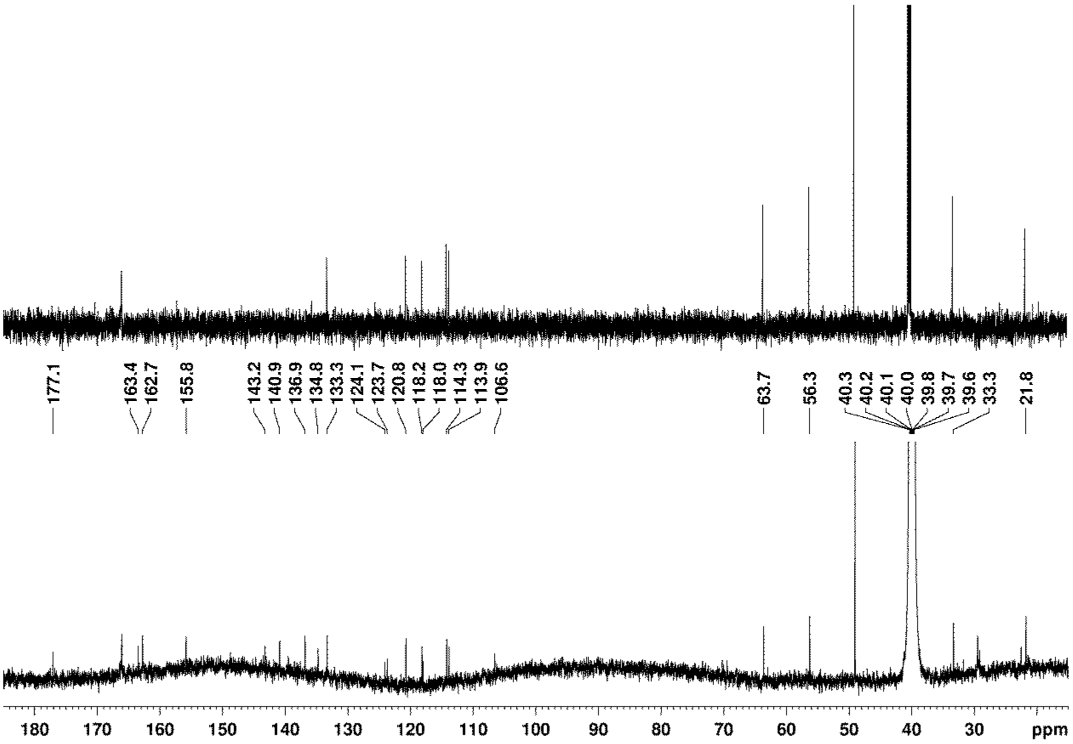

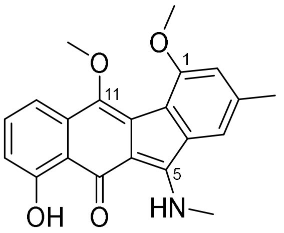

trimethylstealthin C (**13**)

Figure S32. The ^1H-^1H COSY spectrum of trimethylstealthin C (**13**) in DMSO-d_6

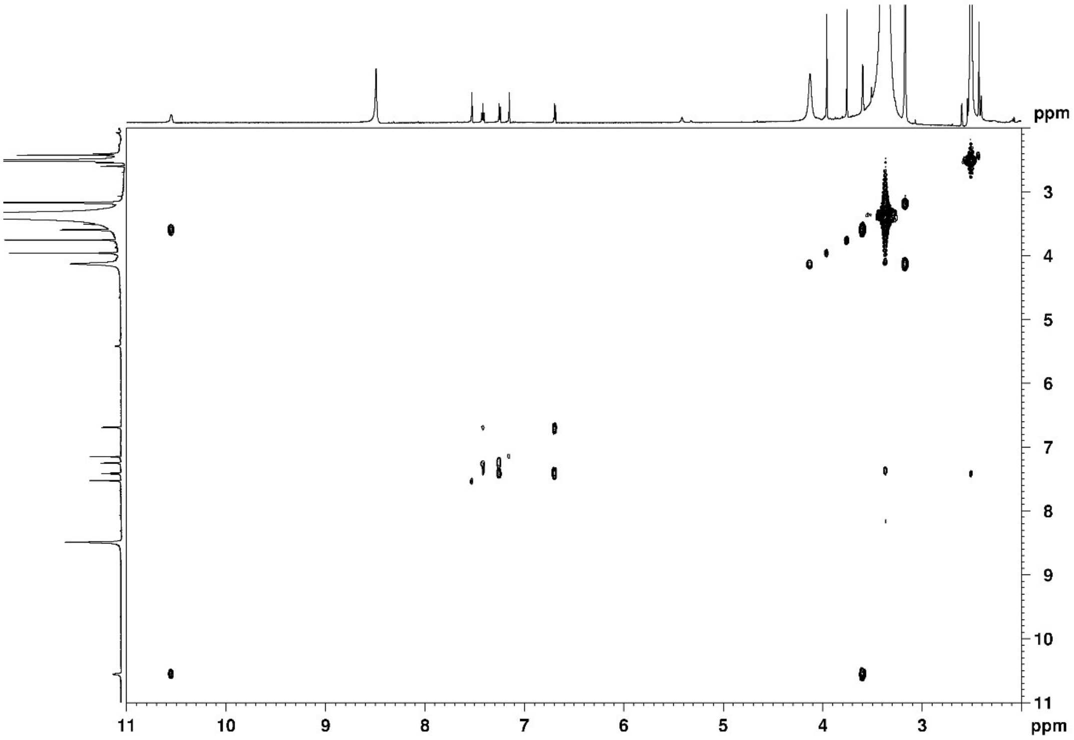

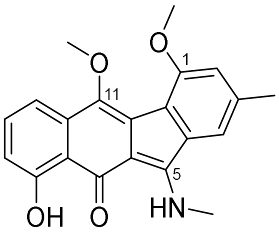

trimethylstealthin C (**13**)

Figure S33. The HSQC spectrum of trimethylstealthin C (**13**) in DMSO-d_6

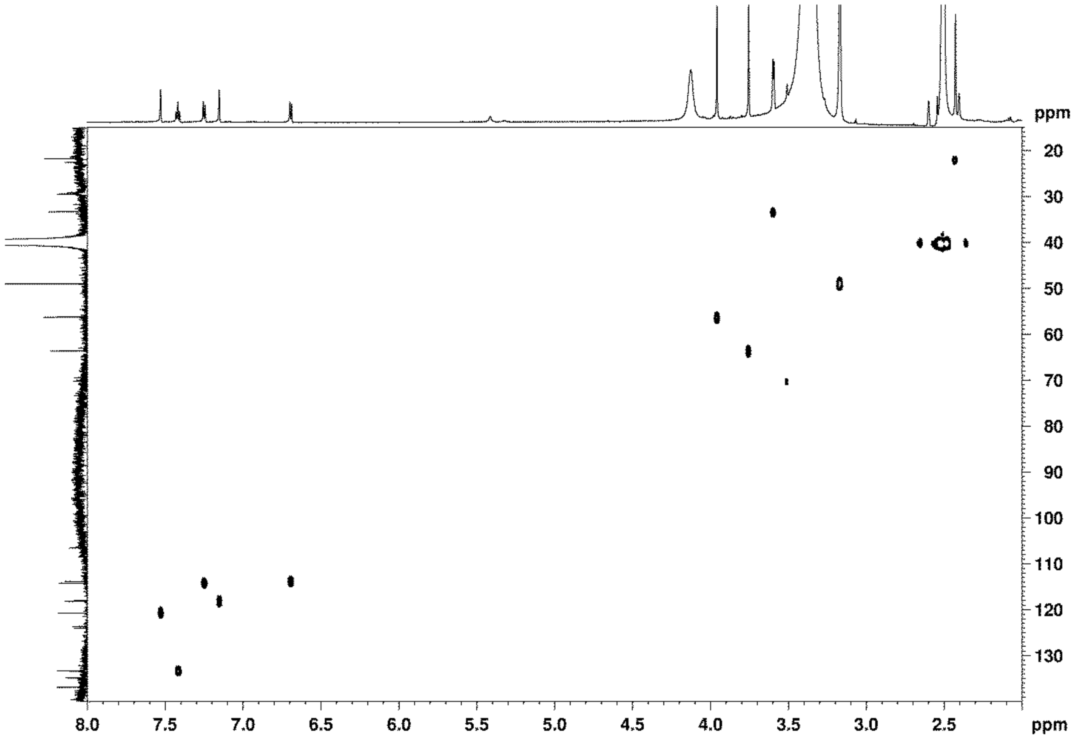

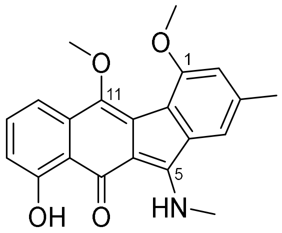

trimethylstealthin C (**13**)

Figure S34. The HMBC spectrum of trimethylstealthin C (**13**) in DMSO-d_6

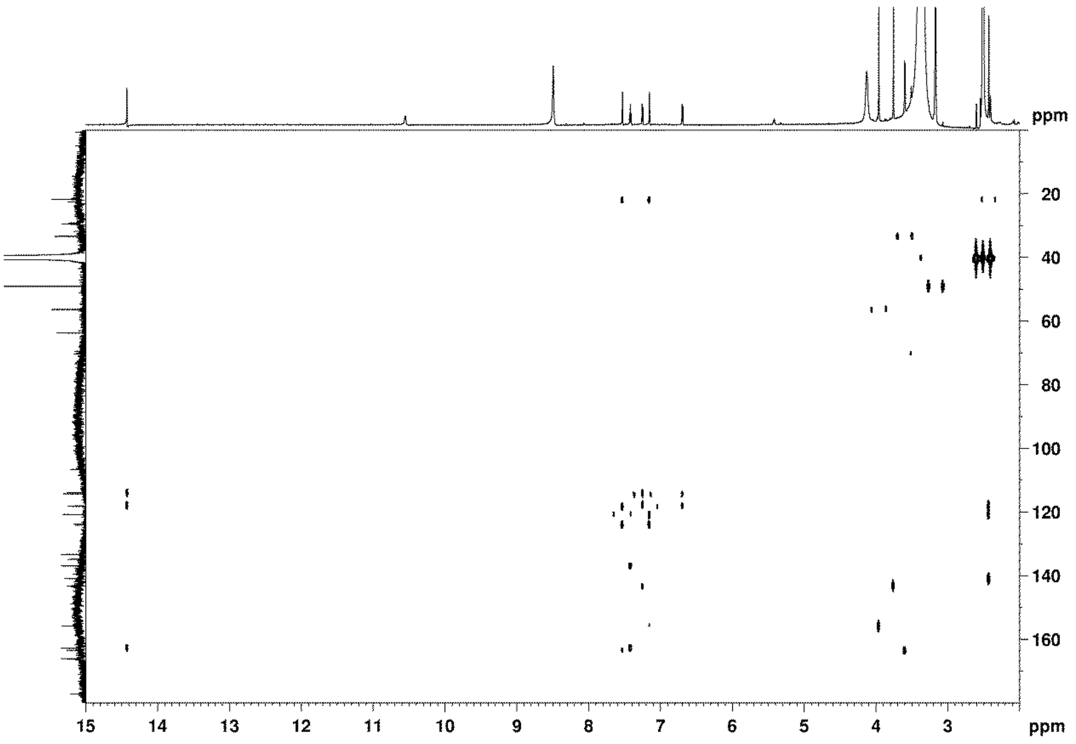

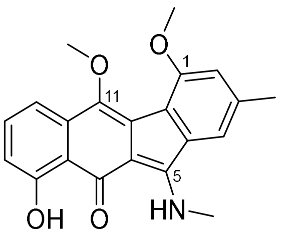

trimethylstealthin C (**13**)

Table S1. Strains, plasmids and primers used in this study

**Strains/Plasmids**	**Characteristic(s)**	**Sources**
E.coli		
BW25113	Host strain for PCR targeting	[1]
ET12567	Donor strain for conjugation	[2]
DH5α	Host strain for cloning	Invitrogen
M. rosaria SCSIO N160	The producing strain of fluostatins	
FLS23	A mutant of *M. rosaria* SCSIO N160 where the *flsN3* gene was inactivated	This study
**Plasmids**		
pUZ8002	Km^r, includeing *tra* for conjugation	[3]
pIJ773	Apr^r, source of *aac(3)IV*	
pCSG5001	A cosmid of SuperCos1-based genomic library of strain SCSIO N160	
pCSG5028	pCSG5001 derivative where *flsN3* was replaced with *aac(3)IV* by insertional mutagenesis	This study
**Primers**	**Sequences**	
**For *flsN3* disruption and confirmation of mutants' genotype**		
flsN3DF	AAGGCCATCGCCGAGCGCGATCCGGCCCTGCGTGCCTTCattccggggatccgtcgacc	
flsN3DR	CAGCAACTGGTCGCCGCCGGCCGGCCCGACCAACTGGCCtgtaggctggagctgcttc	
flsN3DTF	CGGTGGAAGGAATGCCCGTT	
flsN3DTR	CGGGTGAACATGTCGACATC	

Table S2. Crystal data and structure refinement for stealthin D (**9**)

Identification code	1887925
Empirical formula	$C_{21}H_{17}NO_5$
Formula weight	363.36
Temperature/K	100.00 (10)
Crystal system	triclinic
Space group	P-1
a/Å	7.77070(10)
b/Å	8.6632(2)
c/Å	13.5470(2)
α/°	92.2060(10)
β/°	103.699(10)
γ/°	109.139(2)
Volume/Å^3	830.33(3)
Z	2
ρ_{calc}g cm^{-3}	1.453
μ/mm^{-1}	0.865
F(000)	380.0
Crystal size/mm^3	0.2 × 0.1 × 0.1
Radiation	Cu Kα (λ = 1.54184)
2Θ range for data collection/°	10.898 to 148.476
Index ranges	$-9 \leq h \leq 9$, $-10 \leq k \leq 10$, $-16 \leq l \leq 15$
Reflections collected	17405
Independent reflections	3294 [R_{int} = 0.0272, R_{sigma} = 0.0182]
Data/restraints/parameters	3294/0/248
Goodness-of-fit on F^2	1.057
Final R indexes [I>=2σ (I)]	R_1 = 0.0359, wR_2 = 0.0971
Final R indexes [all data]	R_1 = 0.0383, wR_2 = 0.0990
Largest diff. peak/hole / e Å^{-3}	0.20/-0.26

Table S3. Crystal data and structure refinement for stealthin F (**11**)

Identification code	1887926
Empirical formula	$C_{22}H_{19}NO_5$
Formula weight	377.38
Temperature/K	150.00 (10)
Crystal system	triclinic
Space group	P-1
a/Å	9.7527(2)
b/Å	14.4380(2)
c/Å	26.1676(4)
α/°	77.566(10)
β/°	83.3470(10)
γ/°	86.362(10)
Volume/Å^3	3571.17(11)
Z	8
ρ_{calc}g/cm^3	1.404
μ/mm^{-1}	0.825
F(000)	1584.0
Crystal size/mm^3	0.45 × 0.3 × 0.03
Radiation	Cu Kα (λ = 1.54184)
2Θ range for data collection/°	6.956 to 149.474
Index ranges	$-12 \leq h \leq 11$, $-18 \leq k \leq 18$, $-32 \leq l \leq 32$
Reflections collected	66899
Independent reflections	14380 [R_{int} = 0.0294, R_{sigma} = 0.0198]
Data/restraints/parameters	14380/0/1049
Goodness-of-fit on F^2	1.034
Final R indexes [I>=2σ (I)]	R_1 = 0.0398, wR_2 = 0.1048
Final R indexes [all data]	R_1 = 0.0440, wR_2 = 0.1078
Largest diff. peak/hole / e Å^{-3}	0.27/-0.28

References

1. Datsenko, K. A.; Wanner, B. L., One-step inactivation of chromosomal genes in *Escherichia coli* K-12 using PCR products. *Proc. Natl. Acad. Sci. U. S. A.* **2000,** 97, 6640-6645.
2. Macneil, D. J.; Gewain, K. M.; Ruby, C. L.; Dezeny, G.; Gibbons, P. H.; Macneil, T., Analysis of *Streptomyces avermitilis* genes required for avermectin biosynthesis utilizing a novel integration vector. *Gene* **1992,** 111, 61-68.
3. Paget, M. S. B.; Chamberlin, L.; Atrih, A.; Foster, S. J.; Buttner, M. J., Evidence that the extracytoplasmic function sigma factor ς^E is required for normal cell wall structure in *Streptomyces coelicolor* A3(2). *J. Bacteriol.* **1999,** 181, 204-211.
